# Heavy Metal Exposure-Mediated Dysregulation of Sphingolipid Metabolism

**DOI:** 10.3390/antiox13080978

**Published:** 2024-08-12

**Authors:** Shaheer Ahmad, Sierra Single, Yuelong Liu, Kenneth P. Hough, Yong Wang, Victor J. Thannickal, Mohammad Athar, Kayla F. Goliwas, Jessy S. Deshane

**Affiliations:** 1 Department of Medicine, Division of Pulmonary, Allergy, and Critical Care Medicine, University of Alabama at Birmingham, Birmingham, AL 35294-0006, USA; sahmad@uabmc.edu (S.A.); slsingle@uab.edu (S.S.); yuelongliu@uabmc.edu (Y.L.); kenneth@keyqcloud.com (K.P.H.); yongwang@uabmc.edu (Y.W.); 2 John W. Deming Department of Medicine, Tulane University School of Medicine and Southeast Veterans Healthcare System, New Orleans, LA 70119-6535, USA; vthannickal@tulane.edu; 3 Department of Dermatology, University of Alabama at Birmingham, Birmingham, AL 35294, USA; mohammadathar@uabmc.edu

**Keywords:** asthma, heavy metal, oxidative stress, epithelial cells, sphingolipid metabolism

## Abstract

Exposure to heavy metals (HMs) is often associated with inflammation and cell death, exacerbating respiratory diseases including asthma. Most inhaled particulate HM exposures result in the deposition of HM-bound fine particulate matter, PM_2.5_, in pulmonary cell populations. While localized high concentrations of HMs may be a causative factor, existing studies have mostly evaluated the effects of systemic or low-dose chronic HM exposures. This report investigates the impact of local high concentrations of specific HMs (NaAsO_2_, MnCl_2_, and CdCl_2_) on sphingolipid homeostasis and oxidative stress, as both play a role in mediating responses to HM exposure and have been implicated in asthma. Utilizing an in vitro model system and three-dimensional ex vivo human tissue models, we evaluated the expression of enzymatic regulators of the salvage, recycling, and de novo synthesis pathways of sphingolipid metabolism, and observed differential modulation in these enzymes between HM exposures. Sphingolipidomic analyses of specific HM-exposed cells showed increased levels of anti-apoptotic sphingolipids and reduced pro-apoptotic sphingolipids, suggesting activation of the salvage and de novo synthesis pathways. Differential sphingolipid regulation was observed within HM-exposed lung tissues, with CdCl_2_ exposure and NaAsO_2_ exposure activating the salvage and de novo synthesis pathway, respectively. Additionally, using spatial transcriptomics and quantitative real-time PCR, we identified HM exposure-induced transcriptomic signatures of oxidative stress in epithelial cells and human lung tissues.

## 1. Introduction

Environmental exposures in the form of particulate matter (PM) containing heavy metals (HMs) (i.e., cadmium (Cd), arsenic (As), manganese (Mn), and others) represent a major risk factor for pulmonary diseases including asthma and COPD [1,2,3,4]. Inhalation of HM-bound fine PM, PM_2.5_, exposes lung tissue to high concentrations of HMs that underlie the inflammatory response, oxidative stress, and cell death associated with chronic lung diseases [5,6,7]. HMs enriched in PM_2.5_ are not easily degradable, become concentrated, and can cause irreversible damage to human organs and tissues [8,9]. Both adults and children face an elevated asthma risk from HM exposure [2,3,4,10,11]. In experimental models, Cd exposure increased airway reactivity, lung damage, and peribronchiolar fibrosis [12,13,14]. Increased IgE, infiltration of mast and T cells in lungs, coughing, and wheezing mark HM exposure-associated asthma [11,15].

As the first line of defense for air-borne environmental exposures, including HM, the airway epithelium plays a critical role in maintaining the lung microenvironment and orchestrating the inflammatory response. Chronic environmental exposures trigger oxidative stress and initiate signaling pathways associated with airway remodeling in asthma [16,17]. HMs generate reactive oxygen species (ROS) by impairing the antioxidant system and reducing the level of ROS scavengers [18,19,20,21,22]. The accumulation of reactive species may cause lipid peroxidation [23,24], and epidemiological studies link HM exposure to epigenetic changes [25]. Lipids, such as ceramides, prostaglandins, and leukotrienes, are pro-inflammatory mediators of injury and inflammation commonly associated with asthma [26,27,28]. Several significant plasma metabolites and metabolic pathways, including sphingolipid metabolism, have been associated with long-term exposure to ambient PM_2.5_ and ultra-fine particle exposures. These pathways are linked to inflammation, oxidative stress, and overall poor respiratory health across life following exposure [29,30]. A potential role has also been identified for sphingolipids in mediating the proatherogenic effects of short- and medium-term exposure to PM_2.5_ [31]. In murine models of PM_2.5_ exposure, dysregulated hepatic phospholipid metabolism, with increased glycerophospholipid synthesis and disturbed sphingolipid metabolism, has been reported [32]. In general, HM exposures are known to cause dysregulation of lipid metabolism [33,34,35], including in the airways of individuals with asthma [36,37,38]. While altered sphingolipid homeostasis has been associated with the persistent inflammatory response in asthma [39,40,41,42,43,44], the direct effects of high-dose HM exposure on lung tissues and lung epithelium, equivalent to PM_2.5_, have not yet been explored.

Anti-apoptotic lipids like sphingosine-1-phosphate (S1P) and dihydrosphingosine-1-phosphate (DHS1P) stimulate growth and suppress apoptosis, while pro-apoptotic lipids ceramides (Cer), sphingosine (So), dihydrosphingosine (DHSo), sphingomyelin (SM), and monohexosylceramides promote apoptosis and inhibit proliferation [45]. Cell fate decisions are partially regulated by the balance of anti-apoptotic sphingolipids, S1P, and pro-apoptotic sphingolipids, Cer, referred to as the “sphingolipid rheostat” [46]. S1P is a critical regulator of cell growth and survival, implicated in both human and mouse asthma models [42,47]. Its crucial role in exacerbating asthma involves enhancing airway constriction, inducing airway hyper-responsiveness, and controlling the function of mast cells, eosinophils, and dendritic cells [39,40,41,42,48,49]. The pro-survival effects of S1P are achieved by inhibiting de novo ceramide synthesis and triggering downstream signaling via transcription factors [49,50], while the pro-proliferative effects are accomplished through its binding to S1P receptors (G protein-coupled receptor) which, in turn, stimulates G1/S progression within the cell cycle, resulting in increased proliferation [49,51]. The pro-apoptotic sphingolipid Cer mediates apoptosis, cellular stress, and sphingolipid metabolism, and is also implicated in asthma [36,37,52].

The cellular sphingolipid pool is preserved through the actions of the salvage, recycling, and de novo pathways. The salvage pathway breaks down complex sphingolipids like SM and glucosylceramide to generate So. Sphingosine (So) can then be recycled to produce Cer [45,53,54]. The salvage pathway plays a vital role in regulating cellular apoptotic responses, growth arrest, cellular signaling, and trafficking, which are the crucial processes that are impaired in asthma [54,55]. Through the recycling pathway, exogenous short-chain Cer are broken down to generate So, which is then reutilized to generate endogenous Cer [45,56]. The activity of the de novo pathway generates Cer, which can be then reutilized to generate complex sphingolipids [45,46,57]. The de novo pathway is also involved in cellular processes that are implicated in asthma [58,59].

Sphingolipid metabolism is regulated by many enzymes and regulatory proteins that generate and maintain the equilibrium of anti-apoptotic and pro-apoptotic sphingolipid metabolites [45,46]. Sphingosine kinase 1 (SPHK1) is involved in sustaining inflammatory states in asthma [41,48,60]. SPHK1 phosphorylates So and DHSo to generate anti-apoptotic S1P and DHS1P [45,49]. SPHK1 also protects cells against apoptosis by inhibiting the mitochondrial or intrinsic death pathway [49]. Environmental diesel exposure increases SPHK1 levels in murine bronchial epithelial cells [38]. Additionally, exposure to allergens induces SPHK1 in mast cells of mice [61]. Ceramide Synthase 2 (CERS2) has an opposing function and uses So to produce pro-apoptotic Cer and offsets the balance of anti-apoptotic S1P. CERS2 null mice are protected from ovalbumin-induced asthma, suggesting a role for CERS2 and Cer in promoting asthma [46,62]. The ORMDL sphingolipid biosynthesis regulator 3 (ORMDL3) is a regulatory binding protein that inhibits the de novo synthesis pathway of sphingolipid metabolism which generates Cer [63]. Several studies have shown that genetic variants of ORMDL3 increase the risk of asthma [64,65]. Moreover, overexpression of ORMDL3 promotes inflammation in human airway epithelial cells [63,66].

In this report, in contrast to existing studies with chronic exposure or systemic exposures with HMs, we aimed to mimic the local high concentration of specific HMs that lung epithelium would encounter due to inhalation and deposition of PM_2.5_ deep in the lower respiratory tract. Therefore, we investigated whether HM exposure at high concentrations modulates the expression of SPHK1, CERS2, and ORMDL3 and alters the abundance and balance of anti-apoptotic and pro-apoptotic sphingolipid metabolites to alter sphingolipid homeostasis, which is critically implicated in asthma. Importantly, we show that in human lung tissues, HM exposure upregulated transcriptomic signatures related to interferon signaling and oxidative stress-induced senescence, whereas genes related to redox signaling, demethylases, and cell cycle regulation were downregulated, and histone-related genes were differentially regulated.

## 2. Materials and Methods

### 2.1. Cell Culture

Bronchial epithelial cells (BEAS-2B) were a kind gift from Dr. Veena Antony (University of Alabama at Birmingham, Birmingham, AL 35233). BEAS-2B cells were originally procured from ATCC (Manassas, VA, USA), Cat no—CRL-3588. BEAS-2B cells were cultured in Bronchial Epithelial Cell Growth Media (Lonza (Norwest, NSW, Australia), Cat no—CC3170 supplemented with growth factors Cat no—CC-41750). BEAS-2B cells were treated with either Sodium Arsenite (NaAsO_2_, Sigma (St. Louis, MO, USA), Cat no—202908), Manganese chloride tetrahydrate (MnCl_2_·4H_2_O Sigma, Cat no—M3634), or Cadmium chloride (CdCl_2_, Sigma Cat no—80299). Molecular grade water (Corning (Corning, NY, USA), Cat no-MT46000Cl) was used as a solvent for metal compounds.

### 2.2. Cell Toxicity Assay (MTT Assay)

BEAS-2B cells were cultured in a 96-well plate at a density of 50 × 10^3^/well and maintained overnight. Cells were exposed to different concentrations of NaAsO_2_ (0.5, 1, 5, 10, 20, 50, and 100 μM), MnCl_2_ (1, 5, 20, 60, 100, 120, and 180 μM) or CdCl_2_ (0.5, 1, 5, 10, 20, 50, and 100 μM) for 24 h. MTT assay was performed according to instructions of the MTT assay kit (Roche (Basel, Switzerland), Cell proliferation Kit I, Cat# 11465007001). The absorbance at 550 nm and 670 nm wavelengths were measured with a spectrophotometer. The relative cell viability (%) related to control was calculated by [OD]test/[OD]control 100%.

### 2.3. Western Blot

Cell and tissue lysates were prepared in RIPA buffer (ThermoFisher, Pierce, Waltham, MA, USA, Ref no—89900), and protein concentrations were quantitated using a Micro BCA Protein assay kit (ThermoFisher, Pierce, Waltham, MA, USA, Cat# 23225). Then, 20–25 µg of lysate were subjected to WB under 12% SDS-PAGE gels to detect the expression of proteins. Gels were transferred overnight onto 0.45 Immobilon-P PVDF membranes (EMD Millipore, Burlington, MA, USA). Blots were blocked in 5% non-fat dry milk (Fisher Scientific, Waltham, MA, USA) for 1 h and incubated with the following primary antibodies: CERS2 (ORIGENE (Rockville, MD, USA), Cat no-TA809918, 1:1500 dilution), ORMDL3 (Sigma, Cat no—ABN417, 1:1500 dilution), and SPHK1 (Cell Signaling (Danvers, MA, USA), Cat no—12071, 1:1500 dilution) overnight at 4 °C and then incubated in secondary anti-Mouse HRP-conjugated (Promega (Madison, WI, USA), Cat no W402B, 1:5000 dilution) or anti-Rabbit HRP-conjugated secondary antibody (Promega, Cat no W4011, 1:5000 dilution). For normalization with loading controls, membranes were probed with GAPDH (Proteintech (San Diego, CA, USA), Cat no—HRP-60004, 1:5000 dilution). Protein bands were detected using a Chemiluminescence substrate (Millipore, WBKLS0500). Densitometry analyses were performed using ImageJ–1.54 software (NIH, Stapleton, NY, USA).

### 2.4. Real-Time PCR

Total RNA from cells was isolated using the Takara isolation kit (NucleoSpin@RNA plus Cat# 740984.50) and reverse transcribed to cDNA using the cDNA synthesis kit (Takara Cat# RR037A, Berkeley, CA, USA) according to the manufacturer’s protocol. A real-time PCR procedure was conducted with TB Green Premix Ex Tag II (Takara Cat# RR820A, USA). The primer sequences used were as follows: SPHK1 (Gene Accession#NM_182965) GCTGGCAGCTTCCTTGAACCAT (Forward), GTGTGCAGAGACAGCAGGTTCA (Reverse), CERS2 (Gene Accession#NM_181746) GCCTTGCTCTTCCTCATCGTTC (Forward), TGCTTGCCACTGGTCAGGTAGA (Reverse), ORMDL3 (Gene Accession#NM_139280) TAACCCACTGGGAGCAGATGG (Forward), CTCATCAGGGACACGGTGTTGA (Reverse), GAPDH (Gene Accession#NM_002046) GTCTCCTCTGAC-TTCAACAGCG (Forward), ACCACCCTGTTGCTGTAGCCAA (Reverse), SMAD4 (Gene Accession#NM_005359) CTACCAGCACTGCCAACTTTCC (Forward), CCTGATGCTATCTGCAACAGTCC (Reverse), Catalase (Gene Accession#NM_001752) GTGCGGAGATTCAACACTGCCA (Forward), CGGCAATGTTCTCAC-ACAGACG (Reverse), FOXO3 (Gene Accession#NM_001455) TCTACGAGTGGATGGTGCGTTG (Forward), CTCTTGCCAGTTCCCTCATTCTG (Reverse) NR3C1 (Gene Accession#NM_000176), GGAATAGGTGCCAAGGATCTGG (Forward), GCTTACATCTGGTCTCATGCTGG (Reverse), H4C12 (Gene Accession# NM_003541) TGCGCGACAATATCCAGGGCAT (Forward), GTTCTCCAGGAACACCTTCAGC (Reverse), MAPKAPK2 (Gene Accession# NM_032960) ACGGTGGAGAACTCTTTAGCCG (Forward), CTTGACATCCCGATGGGCAATG (Reverse), IFN- β (Gene Accession# NM_002176) CTTGGATTCCTACAAAGAAGCAGC (Forward), and TCCTCCTTCTGGAACTGCTGCA (Reverse). Data analysis was performed using the 2^−ΔΔCT^ method.

### 2.5. Cell Viability and Proliferation Assay

PE Annexin V Apoptosis Detection Kit (BD, Cat no-559763) and Fixable Viability Dye (eBioscience (Waltham, MA, USA), eFluor 455UV Cat no—650868) were used to assess apoptotic and overall cell viability in HM-exposed BEAS-2B cells. For PE Annexin V staining, briefly, cells were washed twice with cold PBS and then the cell pellet was re-suspended in 100 μL of 1x Binding. PE Annexin V (5 μL) and 7-AAD (5 μL) were added, and the cells were incubated for 15 min in the dark. For evaluation of cell viability, cells were suspended in 100 μL of PBS, stained with viability dye (1:1000 dilution), and incubated at 4 °C for 30 min. BD Horizon BUV395 Ki67 (Cat# 56401) and eBioscience staining buffer set (Ref # 00-5523-00) were used to detect the presence of cell proliferation. Briefly, cells were washed twice with cold PBS and then the cell pellet was re-suspended in 1 mL of fixation buffer at 4 °C for 45 min. Subsequently, cells were washed twice by adding 2 mL of Permeabilization buffer and re-suspended in 100 μL of buffer, stained with Ki67 antibody (1:100 dilution), and incubated at 4 °C for 30 min in the dark. Fluorescence intensity was evaluated on BD Spectral-Callisto and BD LSR Fortessa. FACS Diva (Version 9.3.1) was used for flow cytometry acquisition of cells, and final data analysis was performed by Flow Jo (Version 10.8, Tree Star, Ashland, OR, USA).

### 2.6. Human Lung (3D)-Perfusion Bioreactor

De-identified, remnant human lung specimens were obtained from lobectomy and wedge resection surgeries performed at the University of Alabama at Birmingham. Uninvolved remnant human lung tissue cores were cultured ex vivo using a three-dimensional (3D)-perfusion bioreactor as described in [67,68]. Briefly, six 3 mm tissue cores were placed within an extracellular matrix (ECM) support (90% collagen type I) (Advanced Biomatrix, Carlsbad, CA, USA) + 10% growth factor reduced Matrigel (Corning, Tewksbury, MA, USA) in the central chamber of a perfusion bioreactor. Following ECM polymerization and through-channel generation, bioreactors were continuously perfused with a defined (serum-free) tissue culture media (50/50 mixture of Bronchial Epithelial Cell Growth Media) (Lonza, Walkersville, MD, USA) and X-Vivo-15 (Lonza). Following an establishment period of three days, tissues were exposed to 20 μM NaAsO_2_, 120 μM of CdCl_2_, or 100 μM of MnCl_2_ for 8 days. Following exposure, tissue cores were split for histologic (formalin-fixed paraffin-embedded) and protein analyses (tissue lysis). Conditioned media (circulating tissue culture media) was collected for sphingolipidomic profiling following the exposure period. This study was approved by the University of Alabama at Birmingham Institutional Review Board (IRB-#300003092) and conducted following approved guidelines and regulations.

### 2.7. Sample Preparation and Extraction of Lipids for Lipidomics Analyses

Untargeted analysis for lipids was performed on cell line culture media or human lung tissues circulating media (normalized to mL media) in 13 × 100 mm borosilicate tubes with a Teflon-lined cap (VWR, catalog no-60827-453, West Chester, PA, USA). A total of 2 mL of methanol was added along with the internal standard cocktail (250 pmol of each species dissolved in a final total volume of 10 μL of ethanol/methanol/water 7:2:1). The contents were dispersed using an ultra sonicator at room temperature for 30 s followed by the addition of 1 mL of CHCl_3_ and test tubes were recapped. This single-phase mixture was incubated at 48 °C overnight. The extract was centrifuged using a tabletop centrifuge, and the supernatant was removed by a Pasteur pipette and transferred to a new tube. The extract was reduced to dryness using a Thermo Speed Vac (Boston Industries, Walpole, MA, USA). The dried residue was reconstituted in 0.5 mL of the starting mobile phase solvent for LC-MS/MS analysis, sonicated for ca 15 s, then centrifuged for 5 min in a tabletop centrifuge before transfer of the clear supernatant to the autoinjector vial for analysis.

### 2.8. LC-MS/MS of Sphingolipids

These compounds were separated by reverse phase LC using a Supelco 2.1 (i.d.) × 50 mm Ascentis Express C18 column (Sigma, St. Louis, MO, USA) and a binary solvent system at a flow rate of 0.5 mL/min with a column oven set to 35 °C. Before injection of the sample, the column was equilibrated for 0.5 min with a solvent mixture of 95% Mobile phase A1 (CH_3_OH/H_2_O/HCOOH, 58/41/1, *v*/*v*/*v*, with 5 mM ammonium formate) and 5% Mobile phase B1 (CH_3_OH/HCOOH, 99/1, *v*/*v*, with 5 mM ammonium formate). After sample injection (typically 40 μL), the A1/B1 ratio was maintained at 95/5 for 2.25 min, followed by a linear gradient to 100% B1 over 1.5 min, which was held at 100% B1 for 5.5 min, followed by a 0.5 min gradient return to 95/5 A1/B1. The column was re-equilibrated with 95:5 A1/B1 for 0.5 min before the next run. For LC-MS/MS analyses, a Shimadzu Nexera LC-30 AD binary pump system coupled to a SIL-30AC autoinjector and DGU20A_5R_ degasser coupled to an AB Sciex 5500 quadrupole/linear ion trap (QTrap) (SCIEX, Framingham, MA, USA) operating in a triple quadrupole mode was used. Q1 and Q3 were set to pass molecularly distinctive precursor and product ions (or a scan across multiple *m*/*z* in Q1 or Q3), using N_2_ to collisionally induce dissociations in Q2 (which was offset from Q1 by 30–120 eV). The ion source temperature was set to 500 °C.

### 2.9. Immunofluorescent Staining

Ex vivo cultured tissues were formalin-fixed paraffin-embedded (FFPE) and a tissue microarray (TMA) was generated for histologic analysis using 2 mm tissue cores (2 cores per sample). Immunofluorescent staining was performed to detect SK1 (1:200; LS Bio, Seattle, WA, USA), Pan-cytokeratin (1:250; clone: AE-1/AE-3; Novus Biologics, Centennial, CO, USA) and CD45 (1:500; clone:HI30; Biolegend, San Diego, CA, USA) in 5-micron sections following antigen retrieval (10 mM citrate buffer (pH 6, Biogenex, San Ramon, CA, USA)), permeabilization, and blocking. Simultaneous incubation of SPHK1 and CD45 or Pan-cytokeratin was completed before secondary staining with anti-rabbit AlexaFluor-594 (recognizing SPHK1, 1:500, Invitrogen, Waltham, MA, USA) and anti-mouse AlexaFluor-647 (recognizing CD45, 1:500, Invitrogen) or anti-mouse AlexaFluor-488 (recognizing Pan-cytokeratin, 1:500, Invitrogen) and DAPI (1:1000; BD Biosciences, Franklin Lakes, NJ, USA) counterstaining. A Nikon A1R confocal microscope (Plan Fluor 10x DIC L N1 dry 20x 0.75 NA) with Nis Elements 5.0 Imaging Software was used to acquire representative photomicrographs.

### 2.10. Measurement of Cellular Oxidative Stress

Following HM exposure as described above, BEAS-2B cells were labeled with CellROX (molecular probes life technologies, Cat. No—C10448) following the manufacturer’s instructions. Briefly, cells were incubated with 1 µM CellROX dye for 30 min at 37 °C in the dark. Following incubation, cells were washed twice with pre-warmed PBS. CellROX signal was detected by using a detector wavelength of (485/520 nm). Fluorescence intensity was evaluated on BD LSR Fortessa. FACS Diva (Version 9.3.1) was used for the flow cytometry acquisition of cells, and the final data analysis was performed by Flow Jo (Version 204 10.8, Tree Star, USA).

### 2.11. GeoMx Digital Spatial Profiling

Spatial transcriptomic profiling was completed on the TMA described above using the GeoMx Digital Spatial Profiler (DSP) (DSP-01-01, VHDX Version 3.1.0.6) with the GeoMx Human Whole Transcriptome Atlas (Nanostring Technologies, Seattle, WA, USA). Briefly, a FFPE TMA section was incubated overnight at 38 °C followed by an additional 2 h at 60 °C. The section was then manually stained with the Human Whole Transcriptome Atlas UV-cleavable barcoded RNA probes along with antibodies against human Pan-cytokeratin and CD45, as well as SYTO 13 for geometric region of interest (ROI) selection. Using the GeoMx DSP, ROIs were selected, and barcoded RNA probes were cleaved and collected from each ROI. Library Prep with Seq Code primers was performed and the library was sequenced on an Illumina NovaSeq 6000 sequencing instrument. FASTQ files were then converted into digital count conversion (DCC) files using GeoMx NGS Pipeline and uploaded onto the GeoMx Analysis Platform. Data then underwent quality control and Q3 normalization prior to analysis. Clustered heatmaps were generated and unpaired Student’s *t*-tests with Benjamini–Hochberg correction were performed to compare HM to vehicle control. Volcano plots were generated to show the measure of significance (−log10 of *p*-values) vs. the difference in geometric means of probe expression between HM and vehicle control. Additionally, untargeted Gene Ontology analysis and targeted Gene Set Enrichment analysis were completed in R (version 4.3.2).

### 2.12. Statistical Analysis

Statistical analyses were performed using GraphPad Prism -10 software (La Jolla, CA, USA), the GeoMx Analysis Platform, or R. Data are presented as mean ± standard deviation unless indicated otherwise in the figure legend. A *p*-value less than 0.05 was considered to be statistically significant. A two-tailed unpaired Student’s *t*-test was performed to compare the two groups. One-way ANOVA with Tukey’s multiple comparisons test, Bartlett’s multiple comparisons test, or two-way ANOVA with Sidak’s multiple comparison test was performed to compare data with more than two groups to determine statistical significance.

## 3. Results

### 3.1. Exposure to a High Dose of HM Induces Viability Changes in a Dose-Dependent Manner in Lung Epithelial Cells

**Figure 1:**
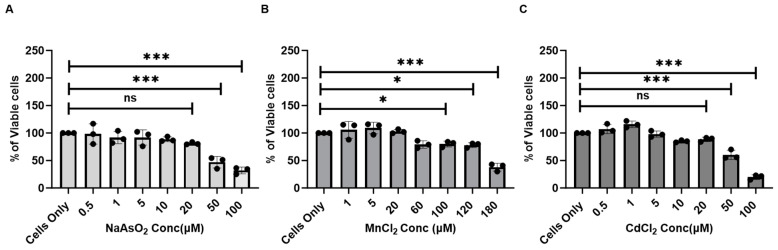
HM exposure-mediated cell cytotoxicity in lung epithelial cells. (**A**) MTT assay performed on BEAS-2B cells exposed to different NaAsO_2_ doses (0, 0.5, 1, 5, 10, 20, 50, and 100 µM). (**B**) MTT assay performed on BEAS-2B cells exposed to different MnCl_2_ doses (0, 1, 5, 20, 60,100, 120, and 180 µM). (**C**) MTT assay performed on BEAS-2B cells exposed to different CdCl_2_ doses (0.5, 1, 5, 10, 20, 50, and 100 µM). One-way ANOVA with Bartlett’s multiple comparisons test was performed to determine the statistical significance at alpha 0.05. Each bar represents the mean ± SD of 3 independent experiments. * *p* < 0.05, *** *p* < 0.001, ns (not significant).

Exposure to HM is associated with the alteration of various cellular signaling pathways in lung epithelial cells. We chose to investigate the impact of increasing HM (arsenic, manganese, and cadmium) concentrations on lung epithelial cells, drawing upon previous findings [13,69,70,71,72,73]. We first investigated if HM exposure in a specific concentration range could induce cellular cytotoxicity. Lung epithelial (BEAS-2B) cells were exposed to increasing concentrations of NaAsO_2,_ MnCl_2_, and CdCl_2_ (0.5–100 µM), and changes in viability were assessed following 24 h exposure. We observed that high-dose exposures of NaAsO_2_ (above 20 µM), MnCl_2_ (above 120 µM), and CdCl_2_ (above 20 µM) decreased cell viability in a dose-dependent manner (Figure 1A–C).

### 3.2. Exposure to HM Alters Sphingolipid Metabolic Pathways in a Dose-Dependent Manner in Lung Epithelial Cells

We next investigated if sub-toxic exposure alters the expression of sphingolipid metabolic enzymes by determining the expression of SPHK1, CERS2, and ORMDL3 in BEAS-2B cells exposed to different doses of NaAsO_2_, MnCl_2_, CdCl_2,_ and untreated controls at 24 h. In NaAsO_2_ (1 µM, 10 µM, or 20 µM)-exposed BEAS-2B cells, expression of SPHK1 was upregulated in a dose-dependent manner (Supplementary Figure S1A,B), whereas the expression of CERS2 and ORMDL3 were not altered compared to controls (Supplementary Figure S1A,C,D). Similarly, in BEAS-2B cells exposed to sub-toxic (20 µM, 60 µM, or 120 µM) doses of MnCl_2_, expression of SPHK1 was upregulated in a dose-dependent manner (Supplementary Figure S2A–C), whereas expression of CERS2 and ORMDL3 was not altered compared to controls (Supplementary Figure S2A,D). The expression of these enzymes did not significantly differ between exposure to 100 µM or 120 µM of MnCl_2_ (Supplementary Figure S2E,H). Furthermore, following sub-toxic exposure of CdCl_2_ (1 µM, 5 µM, and 10 µM), expression of SPHK1, CERS2, and ORMDL3 remained unchanged compared to controls (Supplementary Figure S3A–D).

### 3.3. Exposure to HM Induces Time-Dependent Changes in the Sphingolipid Metabolic Pathways in Lung Epithelial Cells

**Figure 2:**
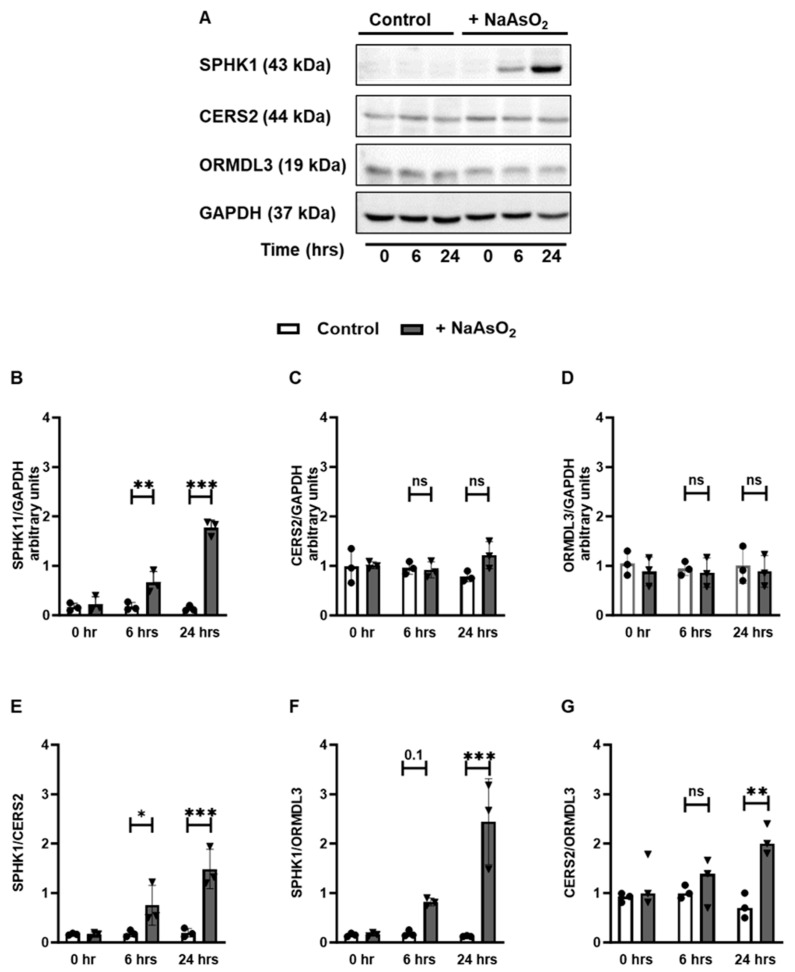
Alteration in sphingolipid metabolic pathways in NaAsO_2_-exposed lung epithelial cells. (**A**) Immunoblot analysis of controls and NaAsO_2_-exposed BEAS-2B cells for SPHK1, CERS2, ORMDL3, and GAPDH expression. (**B**–**D**) Densitometry analysis of controls and NaAsO_2_-exposed BEAS-2B cells for SPHK1 expression (**B**), CERS2 expression (**C**), and ORMDL3 expression (**D**). (**E**–**G**) Densitometry analysis of controls and NaAsO_2_-exposed BEAS-2B cells for SPHK1/CERS2 (**E**), SPHK1/ORMDL3 (**F**), and CERS2/ORMDL3 (**G**). Two-way ANOVA with Sidak’s multiple comparisons test was used to compare data between the control and treated groups at different time points. Differences with a *p*-value lower than 0.05 were considered significant. Each bar represents the mean ± SD of 3 independent experiments. * *p* < 0.05, ** *p* < 0.01; *** *p* < 0.001, ns (not significant), closed circles are replicates of controls and closed triangles represent replicates of NaAsO_2_-exposed samples.

We next investigated whether the observed changes in sphingolipid pathway enzymes following NaAsO_2_ and MnCl_2_ exposure were altered in a time-dependent manner. In 20 µM NaAsO_2_-exposed BEAS-2B cells, the expression of SPHK1 was significantly upregulated at 6 and 24 h (Figure 2A,B), whereas the expression of CERS2 and ORMDL3 was not altered compared to controls (Figure 2A,C,D). Furthermore, while the SPHK1/CERS2 ratio increased significantly at 6 and 24 h (Figure 2E), the SPHK1/ORMDL3 and CERS2/ORMDL3 ratios were altered only at 24 h in NaAsO_2_-exposed BEAS-2B cells when compared to controls (Figure 2F,G).

**Figure 3:**
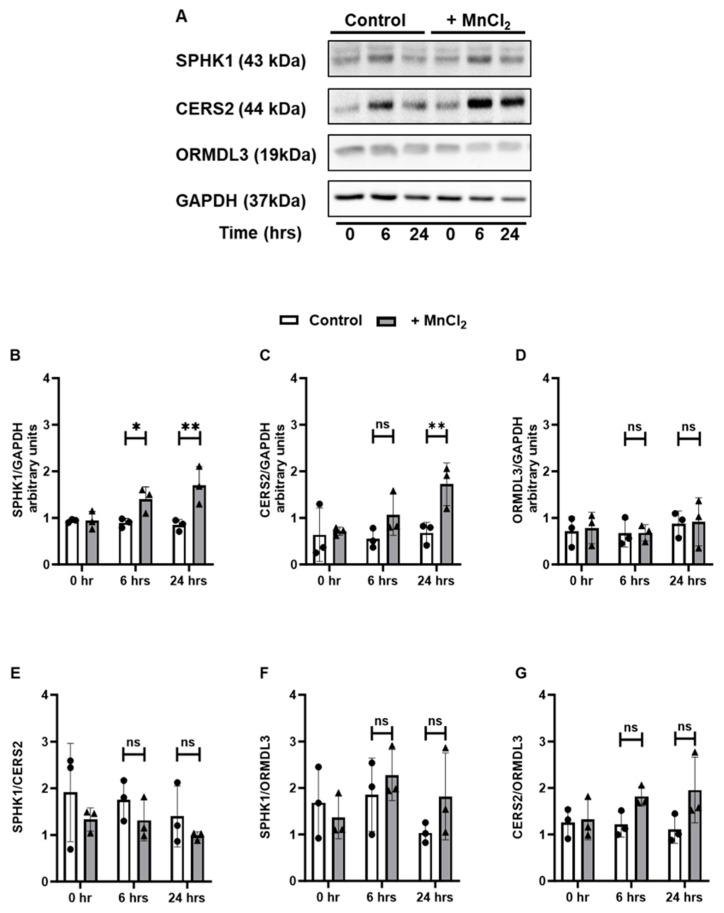
Alteration in sphingolipid metabolic pathways in MnCl_2_-exposed lung epithelial cells. (**A**) Immunoblot analysis of controls and MnCl_2_-exposed BEAS-2B cells for the SPHK1, CERS2, ORMDL3, and GAPDH expression. (**B**–**D**) Densitometry analysis of controls and MnCl_2_-exposed BEAS-2B cells for SPHK1 expression (**B**), CERS2 expression (**C**), and ORMDL3 expression (**D**). (**E**–**G**) Densitometry analysis of controls and MnCl_2_-exposed BEAS-2B cells for the SPHK1/CERS2 (**E**), SPHK1/ORMDL3 (**F**), and CERS2/ORMDL3 (**G**). Two-way ANOVA with Sidak’s multiple comparisons test for data with control and treated group at different time points. Differences with a *p*-value lower than 0.05 were considered significant. Each bar represents the mean ± SD of 3 independent experiments. * *p* < 0.05, ** *p* < 0.01. ns (not significant).

Similarly, in 120 µM MnCl_2_-exposed BEAS-2B cells, expression of SPHK1 was significantly upregulated at 6 and 24 h (Figure 3A,B) and CERS2 was significantly upregulated at 24 h (Figure 3A,C), without any change in ORMDL3 expression at either time point (Figure 3A,D). Interestingly, SPHK1/CERS2, SPHK1/ORMDL3, and CERS2/ORMDL3 ratios were not altered at either 6 or 24 h following MnCl_2_ exposure (Figure 3E–G).

### 3.4. Exposure to HM Alters the Expression of Sphingolipid Metabolizing Enzymes at the Transcript Level

Next, gene expression analyses were completed to determine transcript level changes following HM exposure. Following 24 h NaAsO_2_ exposure, BEAS-2B cell SPHK1 gene expression was significantly higher when compared to controls, while the expression of CERS2 remained unchanged and ORMDL3 expression decreased compared to controls (Supplementary Figure S4A–C). Interestingly, we saw similar differential regulation at the transcript level resulting from MnCl_2_ exposure. While the upregulation of SPHK1 transcripts was noted (Supplementary Figure S4D), the fold change compared to controls was not as robust as with NaAsO_2_ exposure. Additionally, the expression of CERS2 and ORMDL3 decreased significantly in MnCl_2_-exposed BEAS-2B cells compared to controls (Supplementary Figure S4E,F).

### 3.5. HM Exposure Induces Epithelial Cell-Derived Anti-Apoptotic Sphingolipid Metabolites via the Salvage and De Novo Synthesis Pathways

**Figure 4:**
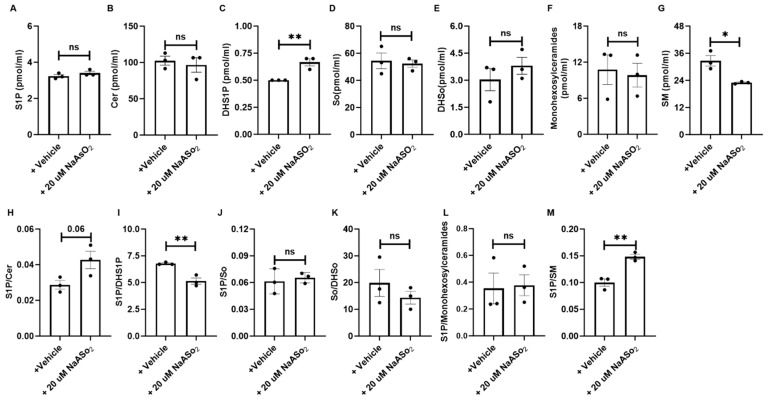
Alteration of sphingolipid metabolites in NaAsO_2_-exposed lung epithelial cells. (**A**–**G**) Levels of S1P (**A**), Cer (**B**), DHSIP (**C**), So (**D**), DHSo (**E**), monohexosylceramides (**F**), and SM (**G**). (**H**–**M**) The ratio of abundance of S1P/Cer (**H**), S1P/DHS1P (**I**), S1P/So (**J**), So/DHSo (**K**), S1P/monohexosylceramides (**L**), and S1P/SM (**M**). A two-tailed unpaired Student’s *t*-test was performed to determine statistical significance at alpha 0.05. Each bar indicates the mean ± SD of 3 independent experiments. * *p* < 0.05, ** *p* < 0.01. ns (not significant).

**Figure 5:**
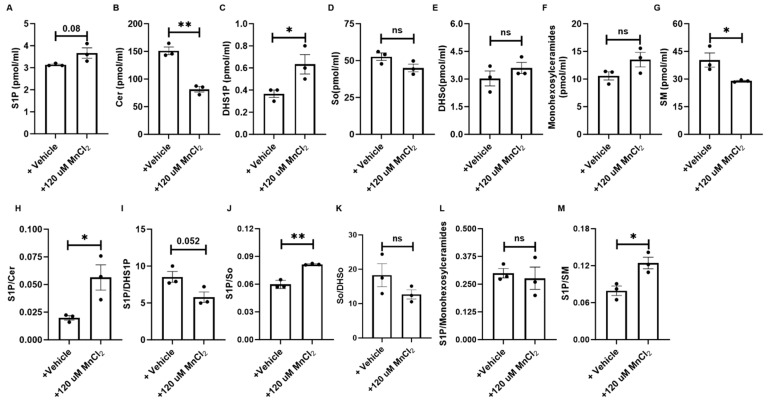
Alteration of sphingolipid metabolites in MnCl_2_-exposed lung epithelial cells. (**A**–**G**) Levels of S1P (**A**), Cer (**B**), DHSIP (**C**), So (**D**), DHSo (**E**), monohexosylceramides (**F**), and SM (**G**). (**H**–**M**) The ratio of abundance of S1P/Cer (**H**), S1P/DHS1P (**I**) S1P/So (**J**), So/DHSo (**K**), S1P/monohexosylceramides (**L**), and S1P/SM (**M**). A two-tailed unpaired Student’s *t*-test was performed to determine statistical significance at alpha 0.05. Each bar depicts the mean ± SD of 3 independent experiments. * *p* < 0.05, ** *p* < 0.01. ns (not significant).

Sphingolipidomic analyses were then performed to quantitate sphingolipid metabolites including anti-apoptotic S1P, DHS1P, and pro-apoptotic Cer, So, DHSo, monohexosylceramides, and SM in conditioned media following HM exposure. As shown in Figure 4A,C, following NaAsO_2_ exposure, the S1P level was not altered; however, the DHS1P level was increased. While SM was reduced (Figure 4G), other metabolites (Cer, So, DHSo, and monohexosylceramides) showed no alteration in the conditioned media of NaAsO_2_-exposed BEAS-2B cells (Figure 4B,D–F). We then assessed whether the sphingolipid rheostat is dysregulated with altered ratios of anti-apoptotic/pro-apoptotic sphingolipid metabolites. As shown in Figure 4H,M, S1P/Cer and S1P/SM increased significantly with NaAsO_2_ exposure, while S1P/So, S1P/monohexosylceramides remain unchanged (Figure 4J,L). Similarly, as shown in Figure 5A,C, in conditioned media of MnCl_2_-exposed BEAS-2B cells, levels of anti-apoptotic S1P, and DHS1P were increased, pro-apoptotic Cer and SM were reduced (Figure 5B,G), while other pro-apoptotic metabolites So, DHSo, and monohexosylceramides did not change (Figure 5D–F). The ratios S1P/Cer, S1P/So, and S1P/SM increased significantly with MnCl_2_ exposure (Figure 5H,J,M), while S1P/monohexosylceramides remained unchanged (Figure 5L). As both HM exposures increased DHS1P levels, this may account for the decreased S1P/DHS1P ratio in HM-exposed BEAS-2B cells (Figure 4I and Figure 5I). Similarly, both HM exposures modestly increased DHSO levels and may account for the decreased So/DHSo ratio (Figure 4K and Figure 5K).

Conversely, in conditioned media of CdCl_2_-exposed BEAS-2B cells, levels of anti-apoptotic S1P, and DHS1P decreased (Supplementary Figure S5A,C), while pro-apoptotic sphingolipids Cer, So, monohexosylceramides, and SM increased (Supplementary Figure S5B,D,F,G), and pro-apoptotic sphingolipid DHSo remained unchanged (Supplementary Figure S5E). When assessing alteration of the sphingolipid rheostat, S1P/Cer, S1P/So, S1P/monohexosylceramides, and S1P/SM were significantly decreased with CdCl_2_ exposure (Supplementary Figure S5H,J,L,M). CdCl_2_ exposure decreased DHS1P levels and this may account for the increased S1P/DHS1P ratio in HM-exposed BEAS-2B cells (Supplementary Figure S5I). Similarly, the unchanged DHSo levels from CdCl_2_ exposure may account for the increased So/DHSo ratio in BEAS-2B cells (Supplementary Figure S5K). 

### 3.6. Exposure to HM Leads to a Limited Induction of Apoptosis in Epithelial Cells

As the sphingolipid rheostat controls apoptosis and survival fate of cells, we assessed if the altered sphingolipid rheostat resulting from HM exposure induced apoptosis in epithelial cells by determining the percentage of late apoptotic/necrotic cells and total cell viability in HM-exposed epithelial cells at 6 and 24 h. The percentage of apoptotic cells was not significantly different in HM-exposed cells compared to controls (Supplementary Figure S6A–D), suggesting minimal induction of apoptosis. This is consistent with reduced levels of pro-apoptotic sphingolipid metabolites following HM exposure. Further, we examined if an increase in the anti-apoptotic S1P or DHS1P, or changes in the ratios of anti-apoptotic/pro-apoptotic sphingolipid metabolites S1P/SM and S1P/Cer, induced proliferation in epithelial cells. The percentage of proliferating Ki67^+^ cells was significantly upregulated in HM-exposed cells at 24 h compared to controls (Supplementary Figure S7A,B).

### 3.7. HM Exposure in Human Lung Tissues Alters Expression of the Sphingolipid Enzymatic Pathway

**Figure 6:**
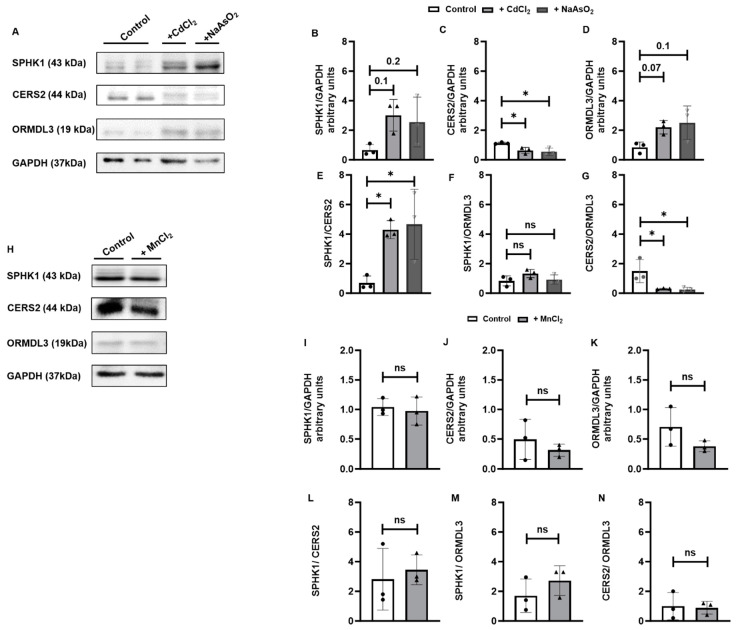
Alteration in sphingolipid metabolic pathways in HM (CdCl_2,_ NaAsO_2,_ and MnCl_2_)-exposed lung tissues. (**A**) Immunoblot analysis of controls, CdCl_2,_ and NaAsO_2_-exposed lung tissues for the SPHK1, CERS2, ORMDL3, and GAPDH expression. (**B**–**D**) Densitometry analysis of controls and CdCl_2_ and NaAsO_2_-exposed lung tissues for SPHK1 expression (**B**), CERS2 expression (**C**), and ORMDL3 expression (**D**). (**E**–**G**) Densitometry analysis of controls, CdCl_2_ and NaAsO_2_-exposed lung tissues for the SPHK1/CERS2 (**E**), SPHK1/ORMDL3 (**F**), and CERS2/ORMDL3 (**G**). (**H**) Immunoblot analysis of controls and MnCl_2_-exposed lung tissues for SPHK1, CERS2, ORMDL3, and GAPDH expression. (**I**–**K**) Densitometry analysis of controls and MnCl_2_-exposed lung tissues for SPHK1 expression (**I**), CERS2 expression (**J**), and ORMDL3 expression (**K**). (**L**,**N**) Densitometry analysis of controls and MnCl_2_-exposed lung tissues for the SPHK1/CERS2 (**L**), SPHK1/ORMDL3 (**M**), and CERS2/ORMDL3 (**N**).Two-way ANOVA with Sidak’s multiple comparisons test for data with more than two groups was performed to determine statistical significance and a two-tailed unpaired Student’s *t*-test for data with two groups was performed to determine statistical significance at alpha 0.05. Each bar represents the mean ± SD of 3 independent experiments. * *p* < 0.05. ns (not significant).

To validate the HM-induced alteration of sphingolipid metabolism in the human lung, we investigated the expression of SPHK1, CERS2, and ORMDL3 in HM-exposed human lung tissues, with HM exposure dose based on prior reports [74,75]. Remnant uninvolved lung tissue cores were cultured using a perfusion bioreactor platform and exposed to 100 µM CdCl_2,_ 20 µM NaAsO_2,_ and 100 µM MnCl_2_. As shown in Figure 6A,B,D, the expression of SPHK1 and ORMDL3 was modestly increased following CdCl_2_ and NaAsO_2_ exposure, while CERS2 expression decreased significantly (Figure 6A,C). As CERS2 expression was reduced in CdCl_2_ and NaAsO_2_-exposed lung tissues, the SPHK1/CERS2 ratio increased with these HM exposures compared to controls (Figure 6E). Similarly, as ORMDL3 expression modestly increased with CdCl_2_ and NaAsO_2_, the CERS2/ORMDL3 ratio decreased with these exposures compared to controls (Figure 6G). Interestingly, the expression of these enzymes in lung tissues was not altered following MnCl_2_ exposure (Figure 6H–K). A modest increase in the SPHK1/CERS2 ratio was seen in MnCl_2_-exposed lung tissues (Figure 6L), while the other ratios remained unchanged compared to controls (Figure 6M,N).

### 3.8. Inhibition of De Novo Synthesis and Induction of Salvage Pathway-Mediated Regulation of Sphingolipid Metabolism in HM-Exposed Lung Tissues

**Figure 7:**
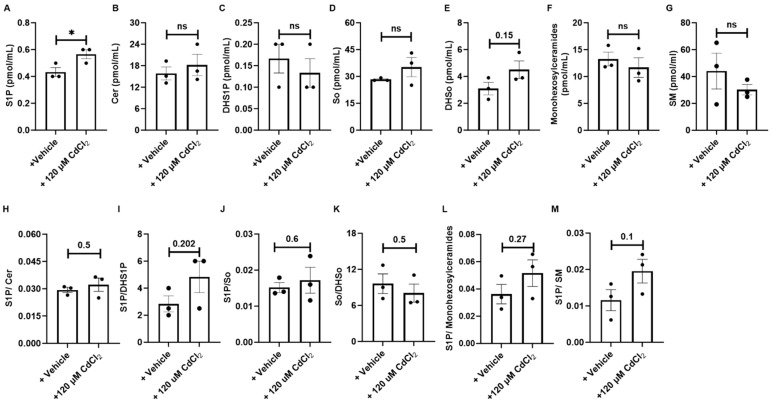
Alteration of sphingolipid metabolites in CdCl_2_-exposed lung tissues. (**A**–**G**) Levels of S1P (**A**), Cer (**B**), DHSIP (**C**), So (**D**), DHSo (**E**), monohexosylceramides (**F**), and SM (**G**). (**H**–**M**) The ratio of abundance of S1P/Cer (**H**), S1P/DHS1P (**I**) S1P/So (**J**), So/DHSo (**K**), S1P/monohexosylceramides (**L**), and S1P/SM (**M**). A two-tailed unpaired Student’s *t*-test was performed to determine statistical significance at alpha 0.05. Each bar indicates the mean ± SD of 3 independent experiments. * *p* < 0.05. ns (not significant).

As HM exposure in human lung tissues differentially altered the expression of sphingolipid pathway enzymes, we performed sphingolipidomics analyses using conditioned media from HM-exposed human lung tissues to measure sphingolipid metabolites. As shown in Figure 7A,C, following CdCl_2_ exposure, anti-apoptotic S1P levels increased significantly, while anti-apoptotic DHS1P was modestly reduced. The levels of pro-apoptotic metabolites So, and DHSo were modestly increased (Figure 7D,E). While little alteration in Cer and monohexosylceramides was observed (Figure 7B,F), SM showed a reduced trend with CdCl_2_ exposure (Figure 7G). We then assessed the alteration of sphingolipid rheostat, and observed that S1P/Cer, S1P/So, S1P/monohexosylceramides, and S1P/SM were modestly increased in CdCl_2_-exposed human lung tissues (Figure 7H,J,L,M).

**Figure 8:**
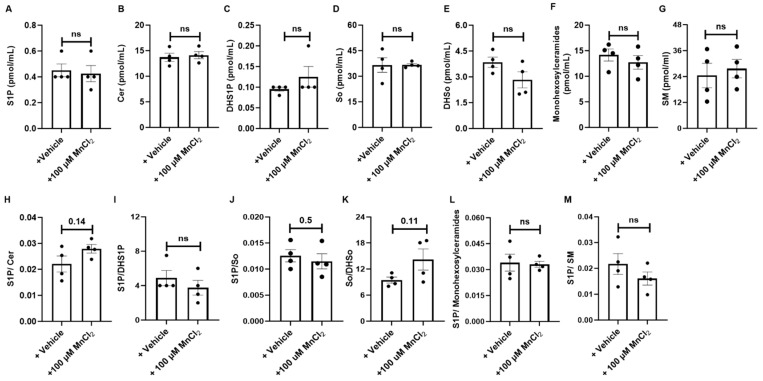
Alteration of sphingolipid metabolites in MnCl_2_-exposed lung tissues. (**A**–**G**) Levels of S1P (**A**), Cer (**B**), DHSIP (**C**), So (**D**), DHSo (**E**), monohexosylceramides (**F**), and SM (**G**). (**H**–**M**) The ratio of abundance of S1P/Cer (**H**), S1P/DHS1P (**I**), S1P/So (**J**), So/DHSo (**K**), S1P/monohexosylceramides (**L**), and S1P/SM (**M**). A two-tailed unpaired Student’s *t*-test was performed to determine statistical significance at alpha 0.05. Each bar indicates the mean ± SD of 3 independent experiments. ns (not significant).

Contrary to what was observed with CdCl_2_ exposure, MnCl_2_ exposure did not alter the levels of any of these sphingolipid metabolites (Figure 8A–D,F,G), except for a modest reduction in DHSo (Figure 8E). We then assessed the alteration of sphingolipid rheostat, and observed only a modest increase in S1P/Cer in MnCl_2_-exposed human lung tissues (Figure 8H). 

Similarly, as shown in Supplementary Figure S8A–G, NaAsO_2_ exposure did not alter sphingolipid metabolites, except for a significant increase in DHS1P in the conditioned media of lung tissue (Supplementary Figure S8C). When evaluating the sphingolipid rheostat, we did not observe changes in S1P/Cer, S1P/So, S1P/monohexosylceramides, or S1P/SM ratios in NaAsO_2_-exposed human lung tissues (Supplementary Figure S8H,J,L,M).

### 3.9. Exposure to HM Leads to Increased Expression of SPHK1 in Epithelial Cells and Other Structural Cells in Human Lung Tissues

**Figure 9:**
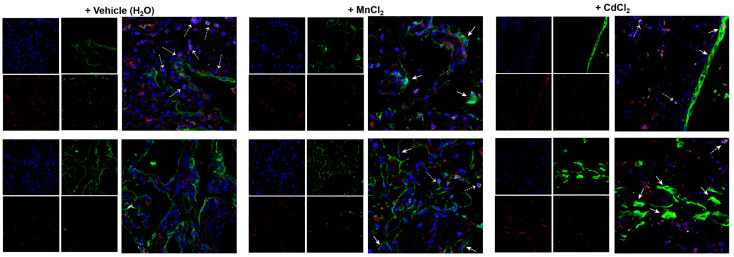
Alteration in sphingolipid metabolic pathways in the epithelial cells of HM (CdCl_2_ and MnCl_2_)-exposed lung tissues. Representative immunofluorescence images of Control, MnCl_2_-exposed, and CdCl_2_-exposed tissue sections showing expression of SPHK1 (red) in PanCK+ epithelial cells (green), CD45+ immune cells (white) and nuclei stain DAPI (blue). White-filled arrows: PanCK+ epithelial cells expressing SK1; White dashed arrows: CD45+ immune cells expressing SK1; grey dotted arrows: SK1+ cells in contact with PanCK+ epithelial cells.

As the regulation of sphingolipid balance observed in HM-exposed epithelial cells was significantly distinct from HM-exposed tissues, we tested whether the tissue microenvironment, particularly the presence of immune cells, may contribute to these alterations following HM exposure. Using immunofluorescence analysis to assess the expression of SPHK1 in epithelial and immune cells in HM-exposed human lung tissues, differential expression of SPHK1 was observed. In HM-exposed tissues, SPHK1 expression was increased in Pan-cytokeratin^+^ (PanCK^+^) epithelial cells, and other structural cells within the tissue in contact with PanCK^+^ epithelial cells also expressed SPHK1. Interestingly, in control tissues, CD45^+^ immune cells were the main cells expressing SPHK1 (Figure 9).

### 3.10. Upregulation of Oxidative Stress with HM Exposure

**Figure 10:**
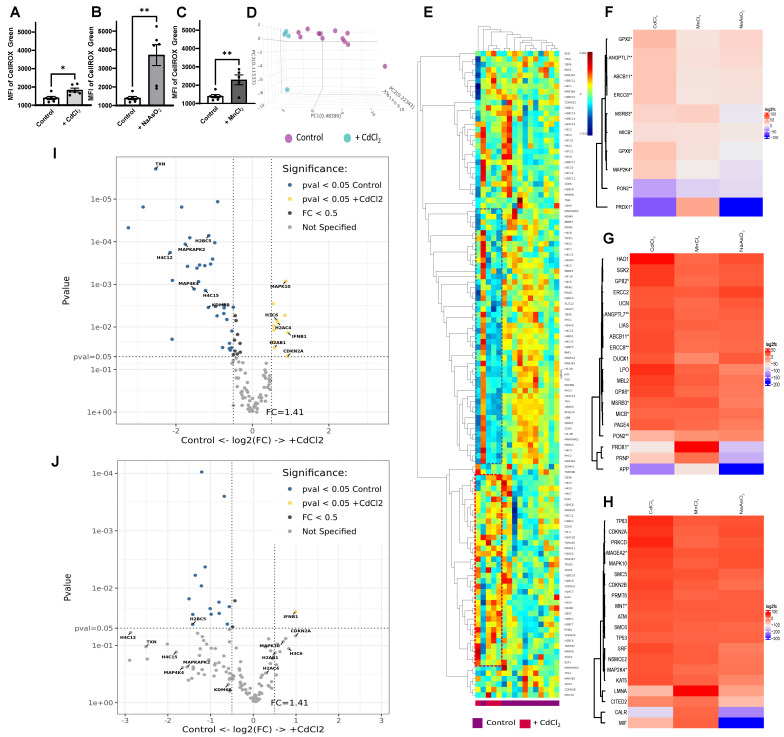
Oxidative Stress following Heavy Metal Exposure. (**A**–**C**) Increased CellROX staining following HM exposure in Beas-2B cells suggests oxidative stress within lung epithelial cells with HM exposure. Each bar represents the mean ± SEM of 2 independent experiments. * *p* < 0.05, ** *p* < 0.01. (**D**) PCA plot of CdCl_2_-exposed and control tissues following spatial transcriptomics shows differential clustering of exposed and control ROIs when Oxidative Stress-Induced Senescence genes were compared. (**E**) Clustered heatmap of Reactome Oxidative Stress-Induced Senescence pathway genes showing ROIs from CdCl_2_-exposed and control tissues. The red box shows genes predominately upregulated with CdCl_2_ exposure and the blue box shows genes predominately downregulated with CdCl_2_ exposure. (**F**–**H**) Targeted GO pathway analyses comparing 3 metal exposures showing common differentially regulated genes across exposures when assessing Oxidative Stress and Senescence Pathways (**F**), Oxidative Stress Pathway alone (**G**), and Senescence Pathway alone (**H**); * *p* < 0.05 and ** *p* < 0.01. (**I**,**J**) Volcano plots showing differentially regulated genes following CdCl_2_ exposure when compared to all controls (**I**) or tissue-matched controls (**J**).

As aberrant sphingolipid signaling has been linked to oxidative stress [76], we next evaluated whether changes in oxidative stress were found with HM exposure. When BEAS-2B cells were exposed to HM_,_ a significant increase in total cellular ROS was observed when compared to vehicle-exposed cells (Figure 10A–C). Using Nanostring GeoMx Digital Spatial Profiling, we then assessed oxidative stress-related pathways in HM-exposed lung tissues, and differences in Oxidative Stress-Induced Senescence and FOXO Mediated Oxidative Stress Reactome pathways were observed with HM treatment. When the top 20 upregulated and downregulated Oxidative Stress Induced Senescence genes were evaluated, 17 of the upregulated and 16 of the downregulated genes were common between at least two HM exposures, with 9 upregulated and 7 downregulated genes common to all three HM exposures (Supplementary Table S1). Similarly, of the top 10 upregulated and downregulated FOXO Mediated Oxidative Stress genes, 8 of the upregulated and 10 of the downregulated genes were common between at least two HM exposures, with 5 upregulated and 6 downregulated genes common to all three HM exposures (Supplementary Table S2). Regions of interest (ROIs) from CdCl_2_-exposed tissues clustered separately from controls when principal component analysis (PCA) was completed using the Oxidative Stress-Induced Senescence gene set (Figure 10D). Similar results were observed when using the FOXO Mediated Oxidative Stress gene set (Supplementary Figure S9A). The clustered heatmap analysis shows specific gene subsets that are upregulated or downregulated within each Reactome pathway (Figure 10E, Supplementary Figures S9B–D and S10A,B). When targeted Gene Ontology (GO) pathway analyses were completed to compare gene expression changes between the three HM exposures, common differentially regulated genes across all exposures were found when assessing Oxidative Stress and Senescence Pathways (Figure 10F), Oxidative Stress Pathway alone (Figure 10G), and Senescence Pathway alone (Figure 10H). Genes related to interferon signaling (IFNB1) and senescence (CDKN2A) were upregulated, whereas genes related to redox signaling (TXN), demythlases (KDM6B), and cell cycle regulation (UBA52) were downregulated. MAPK pathway (upregulated: MAP2K6 and MAPK10; downregulated: MAP4K4 and MAPKAPK2) and histone-related genes (upregulated: H2AC4, H2AB1, H3C6, etc.; downregulated: H4C12, H4C15, H2BC5, etc.) were differentially regulated, as shown in Figure 10I and Supplementary Figures S9A–D and S10C,D. Volcano plots show similar up and downregulated genes when comparing CdCl_2_-exposed tissue to all controls or tissue-matched controls (Figure 10I,J). In untargeted Gene Set Enrichment (GSE) analysis, redoxin and oxidase pathways were found to be suppressed following HM exposure (Supplementary Figure S11). These results were confirmed by evaluation of the SMAD4, Catalase, FOXO3, and NR3C1 transcript levels in HM-exposed BEAS-2B cells. Following 24 h exposure of BEAS-2B cells to NaAsO_2_ (20 µM), SMAD4, and Catalase, gene expression was significantly reduced. In contrast, the expression of FOXO3 and NR3C1 remained unchanged compared to controls (Supplementary Figure S12A–D). Interestingly, the expression of these genes was reduced in BEAS-2B cells exposed to MnCl_2_ (120 µM) and CdCl_2_ (10 µM) (Supplementary Figure S12I–L) compared to controls (Supplementary Figure S12E,H,I–L). Additionally, in HM-exposed BEAS-2B cells, H4C12 expression was significantly reduced, while MAPKAPK2 and IFN-β transcript levels increased compared to controls (Supplementary Figure S13A–I). Together, this data suggests similar increases in oxidative stress following HM exposure in lung epithelial cells and lung tissues.

## 4. Discussion

Environmental exposures, including HM-containing cigarette smoke exposures, are implicated in chronic lung diseases, including asthma and COPD [36,37,38]. Alteration in sphingolipid homeostasis plays a role in sustaining the inflammatory response in asthma [39,40,41,42,43,77]. SPHK1, which regulates the balance of sphingolipid metabolites S1P and Cer, is induced during asthma in human asthmatics and mice with a documented dysregulation of S1P and Cer levels in the context of asthma [42,46,52]. The lungs of allergic asthmatics exhibit increased levels of S1P [36,39]. Anti-apoptotic S1P also eases the contraction of human airway smooth muscle cells, modulates the induction of airway hyper-responsiveness, and controls the activation and function of mast cells, eosinophils, and dendritic cells [39,40,41,48,78]. Here, we report that local high concentrations of HM that mimic the deposition of PM_2.5_ deep in the lower respiratory tract alter sphingolipid homeostasis in both human lung epithelial cells and human lung tissues. The alteration of sphingolipid pathway enzymes in human epithelial cells varied in a dose- and metal-dependent manner.

Differential regulation of sphingolipid metabolism in lung epithelial cells was observed between different metal exposures. With MnCl_2_ exposure, we observed increased expression of SPHK1 and CERS2 and unaltered ORMDL3, as well as increased levels of S1P and DHS1P in BEAS-2B cells. These changes may also reflect persistent activation of both the salvage and de novo pathways. With NaAsO_2_ exposure, we observed increased SPHK1 expression, unaltered CERS2 and ORMDL3, as well as increased DHS1P and reduced SM in BEAS-2B cells. Together, these changes may reflect persistent activation of both the salvage and de novo pathways. Contrary to this observation, we observed increased SM, Cer, So, and monohexosylceramide, and decreased S1P and DHS1P following CdCl_2_ exposure in BEAS-2B cells. These changes suggest activation of the recycling pathway and inactivation of the salvage pathway.

When evaluating responses to HM exposures in lung tissues, similar impacts on the recycling pathway were observed following CdCl_2_ and NaAsO_2_ exposure, with a significant decrease in CERS2 expression coupled with unaltered SM, Cer, So, and monohexosylceramides in these tissues, suggesting that the recycling pathway may be inactive. Differential regulation of the salvage pathway and de novo synthesis pathways were observed between these exposures, with increased SPHK1 and S1P and unchanged DHS1P suggesting activation of the salvage pathway in CdCl_2_-exposed lung tissues, and increased SPHK1 coupled with increased DHS1P and unchanged S1P suggest activation of the de novo synthesis pathway in NaAsO_2_-exposed lung tissues. MnCl_2_-exposed human lung tissues had no alteration in expression of SPHK1, CERS2, and ORMDL3 and an unaltered sphingolipid profile, suggesting inactivation of sphingolipid homeostasis. The altered sphingolipid profiles in HM-exposed tissues were markedly distinct, implying that different HM may utilize different regulatory mechanisms for the modulation of sphingolipid homeostasis. Thus, the activation of both the salvage and de novo synthesis pathways account for dysregulated sphingolipid metabolism in HM-exposed epithelial cells, whereas the activation of either the salvage pathway or the de novo synthesis pathways account for the dysregulated sphingolipid metabolism in HM-exposed human lung tissues. The immune microenvironment and or SPHK1-expressing immune cells in lung tissues may differentially contribute to the regulation of sphingolipid homeostasis in response to HM exposure at the tissue level. The primary responders to HM exposure are the epithelial cells within lung tissues.

In our studies with lung epithelial cells, HMs (NaAsO_2_ and MnCl_2_) modulated the enzyme SPHK1. These observations are consistent with previous reports of upregulation of SPHK1 in murine bronchial epithelial cells exposed to diesel and the induction of SPHK1 in murine mast cells stimulated with allergens [38,61]. In CdCl_2_-exposed human lung tissues, we saw utilization of only the salvage pathway, through SPKH1. Previous studies have associated the de novo pathway with the risk of asthma [43,79]. Further studies are necessary to confirm the activation of both pathways in epithelial injury associated with asthma.

Our findings revealed differential regulation of CERS2 expression with NaAsO_2_, MnCl_2,_ and CdCl_2_ exposure in lung epithelial cells. Despite the increased expression of CERS2 in MnCl_2_ exposures, we observed a reduction in Cer and an increase in the S1P/Cer ratio, which may allow enhanced signaling via S1P. Although regulation of CERS2 by HM exposure has not been previously reported, aerosol administration of ovalbumin increases Cer [52]. Increased airflow resistance and airway inflammation have been reported in CERS2 null mice [62].

ORMDL3 overexpression negatively modulates de novo sphingolipid synthesis and is associated with an increased risk of asthma [63,64,65]. Environmental tobacco smoke upregulates ORMDL3 [80], while low-dose particulate matter exposure increases the expression of the ORMDL3 gene in BEAS-2B cells [81,82]. In our studies with HM-exposed epithelial cells, this was not observed at the protein level; however, we observed a reduction at the transcriptional level. Lack of stability of mRNA may contribute to the lack of induction of protein expression. Additionally, the lack of induction of ORMDL3 expression was aligned with increased de novo synthesis and DHS1P generation.

Our sphingolipidomics analysis revealed a significant difference in the abundance of sphingolipid metabolites when comparing HM-exposed epithelial cells/lung tissues to controls, with a notable increase in anti-apoptotic S1P levels, accompanied by a decrease in pro-apoptotic Cer and SM levels. Despite the limitation of our lipidomics analyses with low sample size and variability, our observations are consistent with secondhand smoke (SHS)-exposed asthmatic individuals exhibiting an increase in S1P levels, and a decline in levels of Cer and SM compared to control groups [36,52].

In our transcriptomic assessment, in addition to the well-established HM-mediated effects on redox regulation of the lung epithelium, we identified modulation of gene signatures consistent with oxidative stress-induced senescence and epigenetic modifications. Additionally, modulation of the FOXO Mediated Oxidative Stress Reactome pathway was identified in HM-exposed lung tissues. FOXO transcription factors are important regulators of the cellular stress response and promote the cellular antioxidant defense [83,84,85,86]. FOXOs are known to stimulate the transcription of antioxidant genes. Reactive oxygen species (ROS), as well as other stressful stimuli that modulate ROS, may alter FOXO activity at multiple levels, including their interaction with coregulators. Moreover, transcriptional and posttranscriptional control of FOXO genes are sensitive to ROS. Although HM-induced lipid peroxidation is well documented, whether HM exposure-induced oxidative stress and senescence are directly associated with dysfunctional sphingolipid metabolism remains to be determined. Nevertheless, these alterations observed in our study reflect the known toxicological effects of HM exposure, their effects on metabolism, and the potential for environmental epigenomics and disease susceptibility. Taken together, our studies show that local high-dose HM exposure in lung epithelial cells and human lung tissues, similar to exposure to PM_2.5_ deep in the lower respiratory tract, alters sphingolipid homeostasis, which is known to play a crucial role in regulating cellular apoptosis, survival, and the activation of immune cells [45,46,49,77]. These observations have important implications for asthma; epithelial injury from environmental exposures and consequent dysregulation of sphingolipid metabolism may exacerbate inflammatory responses and have downstream negative effects on lung function. While our study was limited by the use of a single HM and we did not use PM_2.5_, we were able to determine HM-specific effects on lipid metabolism within human lung epithelial cells and lung tissue. HM exposure modulated the balance of anti-apoptotic and pro-apoptotic sphingolipids in both cells and tissues. Hence, our studies provide insights on the potential contribution of HM environmental exposures to lung injury and inflammation, and how HMs may lead to the development of asthma.

## Data Availability

The datasets generated during and/or analyzed during the current study are available from the corresponding author on reasonable request. Correspondence and requests for materials should be addressed to Jessy Deshane (jessydeshane@uabmc.edu).

## Supplementary Materials

The following supporting information can be downloaded at: https://www.mdpi.com/article/10.3390/antiox13080978/s1, Supplementary Figure S1. Dose-dependent alteration in sphingolipid metabolic pathways in NaAsO_2_-exposed lung epithelial cells. (A) Immunoblot analysis of controls and different doses (1 µM, 10 µM, and 20 µM) of NaAsO_2_-exposed BEAS-2B cells for SPHK1, CERS2, ORMDL3, and GAPDH expression. (B–D) Densitometry analysis of controls and different doses of NaAsO_2_-exposed BEAS-2B cells, illustrating SPHK1 expression (B), CERS2 expression (C), and ORMDL3 expression (D). One-way ANOVA with Tukey’s multiple comparisons test for data with more than two groups was performed to determine the statistical significance at alpha 0.05. Each bar represents the mean ± SD of three independent experiments. * *p* < 0.05, ** *p* < 0.01; *** *p* < 0.001. Supplementary Figure S2. Dose-dependent alteration in sphingolipid metabolic pathways in MnCl_2_-exposed lung epithelial cells. (A) Immunoblot analysis of controls and different doses (20 µM, 60 µM, and 120 µM) of MnCl_2_-exposed BEAS-2B cells for SPHK1, CERS2, ORMDL3, and GAPDH expression. (B-D) Densitometry analysis of controls and different doses of MnCl_2_-exposed BEAS-2B cells illustrating SPHK1 expression (B), CERS2 expression (C), and ORMDL3 expression (D). (E) Immunoblot analysis of controls and different doses (100 µM and 120 µM) of MnCl_2_-exposed BEAS-2B cells illustrating SPHK1, CERS2, ORMDL3, and GAPDH expression. (F–H) Densitometry analysis of controls and different doses of MnCl_2_-exposed BEAS-2B cells for SPHK1 expression (F), CERS2 expression (G), and ORMDL3 expression (H). One-way ANOVA with Tukey’s multiple comparisons test for data with more than two groups was performed to determine the statistical significance at alpha 0.05. Each bar represents the mean ± SD of 3 independent experiments. * *p* < 0.05, ** *p* < 0.01; *** *p* < 0.001. Supplementary Figure S3. Dose-dependent alteration in sphingolipid metabolic pathways in CdCl_2_-exposed lung epithelial cells. (A) Immunoblot analysis of controls and different doses (1 µM, 5 µM, and 10 µM) of CdCl_2_-exposed BEAS-2B cells for SPHK1, CERS2, ORMDL3, and GAPDH expression. (B–D) Densitometry analysis of controls and different doses of CdCl_2_-exposed BEAS-2B cells for SPHK1 expression (B), CERS2 expression (C), and ORMDL3 expression (D). One-way ANOVA with Tukey’s multiple comparisons test for data with more than two groups was performed to determine the statistical significance at alpha 0.05. Each bar represents the mean ± SD of 3 independent experiments. * *p* < 0.05, ** *p* < 0.01; *** *p* < 0.001. Supplementary Figure S4. Alteration in sphingolipid metabolic pathways regulating enzyme transcript levels in HM-exposed lung epithelial cells. (A–C) Fold change in enzyme expression in NaAsO_2_-exposed BEAS-2B cells SPHK1 (A), CERS2 (B), and ORMDL3 (C). (D–F) Fold change in enzyme expression in MnCl_2_-exposed BEAS-2B cells, SPHK1 (D), CERS2 (E), and ORMDL3 (F). A two-tailed unpaired Student’s *t*-test was performed to determine statistical significance at alpha 0.05. Each bar represents the mean ± SD of 3 independent experiments. * *p* < 0.05, ** *p* < 0.01; *** *p* < 0.001. Supplementary Figure S5. Alteration of sphingolipid metabolites in CdCl_2_-exposed lung epithelial cells. (A–G) Levels of S1P (A), Cer (B), DHSIP (C), So (D), DHSo (E), monohexosylceramides (F), and SM (G). (H–M) The ratio of abundance of S1P/Cer (H), S1P/DHS1P (I), S1P/So (J), So/DHSo (K), S1P/monohexosylceramides (L), and S1P/SM (M). A two-tailed unpaired Student’s *t*-test was performed to determine statistical significance at alpha 0.05. Each bar represents the mean ± SD of 3 independent experiments. * *p* < 0.05, ** *p* < 0.01; *** *p* < 0.001. Supplementary Figure S6. Quantitation of apoptosis and cell viability in lung epithelial cells exposed to HMs (MnCl_2_ and NaAsO_2_). (A,B) Representative image of controls and HM-exposed cells for Annexin V^+^ 7AAD^+^ (A), Viability Dye eFlour 455UV^+^ (B). (C,D). Graphical representation of controls and HM-exposed cells for Annexin V^+^ 7AAD^+^ (C) and Viability Dye eFlour 455 UV^+^ (D). Two-way ANOVA with Sidak’s multiple comparisons test for data with more than two groups was performed to determine the statistical significance at alpha 0.05. Each bar represents the mean ± SD of 3 independent experiments. * *p* < 0.05, ** *p* < 0.01; *** *p* < 0.001. Supplementary Figure S7. Quantitation of proliferation of HM (MnCl_2_ and NaAsO_2_) exposed lung epithelial cells. (A) Representative image of controls and HM-exposed cells for Ki67^+^. (B) Graphical representation of controls and HM-exposed cells for Ki67^+^. One-way ANOVA with Tukey’s multiple comparisons test for data with more than two groups was performed to determine the statistical significance at alpha 0.05. Each bar represents the mean ± SD of 3 independent experiments. * *p* < 0.05, ** *p* < 0.01; *** *p* < 0.001. Supplementary Figure S8. Alteration of sphingolipid metabolites in NaAsO_2_-exposed lung tissues. (A–G). Levels of S1P (A), Cer (B), DHSIP (C), So (D), DHSo (E), monohexosylceramides (F), and SM (G). (H–M) The ratio of abundance of S1P/Cer (H), S1P/DHS1P (I), S1P/So (J), So/DHSo (K), S1P/monohexosylceramides (L), and S1P/SM (M). A two-tailed unpaired Student’s *t*-test was performed to determine statistical significance at alpha 0.05. Each bar represents the mean ± SD of 3 independent experiments. * *p* < 0.05, ** *p* < 0.01; *** *p* < 0.001. Supplementary Table S1. Top 20 upregulated and downregulated Reactome Oxidative Stress-Induced Senescence Pathway Genes across HM Exposures. Bold brown text denotes common genes across all three HM exposures, blue bold text denotes common genes with CdCl_2_ and NaAsO_2_ exposure, purple bold text denotes common genes with CdCl_2_ and MnCl_2_ exposure, and bold green text denotes common genes with MnCl_2_ and NaAsO_2_ exposure. Supplementary Table S2. Top 10 upregulated and downregulated Reactome FOXO-Mediated Oxidative Stress Pathway genes across HM exposures. Bold brown text denotes common genes across all three HM exposures, blue bold text denotes common genes with CdCl_2_ and NaAsO_2_ exposure, purple bold text denotes common genes with CdCl_2_ and MnCl_2_ exposure, and bold green text denotes common genes with MnCl_2_ and NaAsO_2_ exposure. Supplementary Figure S9. Reactome FOXO Mediated Oxidative Stress Pathway changes following HM exposure. (A) PCA plot of CdCl_2_-exposed and control tissues following spatial transcriptomics shows differential clustering of exposed and control ROIs when FOXO Mediated Oxidative Stress genes were compared. (B–D). Clustered heatmap of FOXO Mediated Oxidative Stress genes showing ROIs from CdCl_2_-exposed and control tissues (B), NaAsO_2_-exposed and control tissues (C), and MnCl_2_-exposed and control tissues (D). The red box shows genes predominately upregulated with NaAsO_2_ exposure and the blue box shows genes predominately downregulated with NaAsO_2_ exposure. (E–G) Volcano plots showing differentially regulated FOXO Mediated Oxidative Stress genes following CdCl_2_ exposure (E), NaAsO_2_ exposure (F), or MnCl_2_ exposure (G). Supplementary Figure S10. Reactome Oxidative Stress-Induced Senescence Pathway changes following NaAsO_2_ or MnCl_2_ exposure. (A,B) Clustered heatmap of Oxidative Stress-Induced Senescence genes showing ROIs from NaAsO_2_-exposed and control tissues (A), and MnCl_2_-exposed and control tissues (B). (C,D) Volcano plots showing differentially regulated Oxidative Stress-Induced Senescence genes following NaAsO_2_ exposure (C) or MnCl_2_ exposure (D). Supplementary Figure S11: Untargeted Gene Set Enrichment Analysis shows suppressed Redoxin and Oxidase Pathway activity following HM exposure. (A–C). Activated and suppressed Molecular Functions Gene Set Enrichment Analysis following CdCl_2_ (A), NaAsO_2_ (B), or MnCl_2_ (C) exposures when compared to controls. Supplementary Figure S12. Alteration in oxidative stress-regulating enzymes transcript level in HM-exposed lung epithelial cells. (A–D) Fold change in enzyme expression in NaAsO_2_-exposed BEAS-2B cells SMAD4 (A), Catalase (B), FOXO3 (C), and NR3C1 (D). (E–H) Fold change in enzyme expression in MnCl_2_-exposed BEAS-2B cells, SMAD4 (E), Catalase (F), FOXO3 (G), and NR3C1 (H). (I–L) Fold change in enzyme expression in CdCl_2_-exposed BEAS-2B cells, SMAD4(I), Catalase (J), FOXO3 (K), and NR3C1 (L). A two-tailed unpaired Student’s *t*-test was performed to determine statistical significance at alpha 0.05. Each bar represents the mean ± SD of 3 independent experiments. * *p* < 0.05, ** *p* < 0.01; *** *p* < 0.001. Supplementary Figure S13. Alteration in oxidative stress-induced senescence gene expression in HM-exposed lung epithelial cells. (A–C) Fold change in gene expression in NaAsO_2_-exposed BEAS-2B cells H4C12 (A), MAPKAPK2 (B), and IFN-β (C). (D–F) Fold change in gene expression in MnCl_2_-exposed BEAS-2B cells, H4C12 (D), MAPKAPK2 (E), and IFN-β (F). (G–I) Fold change in enzyme expression in CdCl_2_-exposed BEAS-2B cells, H4C12 (G), MAPKAPK2 (H), and IFN-β (I). A two-tailed unpaired Student’s *t*-test was performed to determine statistical significance at alpha 0.05. Each bar represents the mean ± SD of 3 independent experiments. * *p* < 0.05, ** *p* < 0.01; *** *p* < 0.001.
