# Supplementary material for: Heavy Metal Exposure-Mediated Dysregulation of Sphingolipid Metabolism
Source: Antioxidants (Basel). 2024 Aug 12;13(8):978. doi: 10.3390/antiox13080978 (PMC13317051; doi:10.3390/antiox13080978)

Supplementary Figure S1

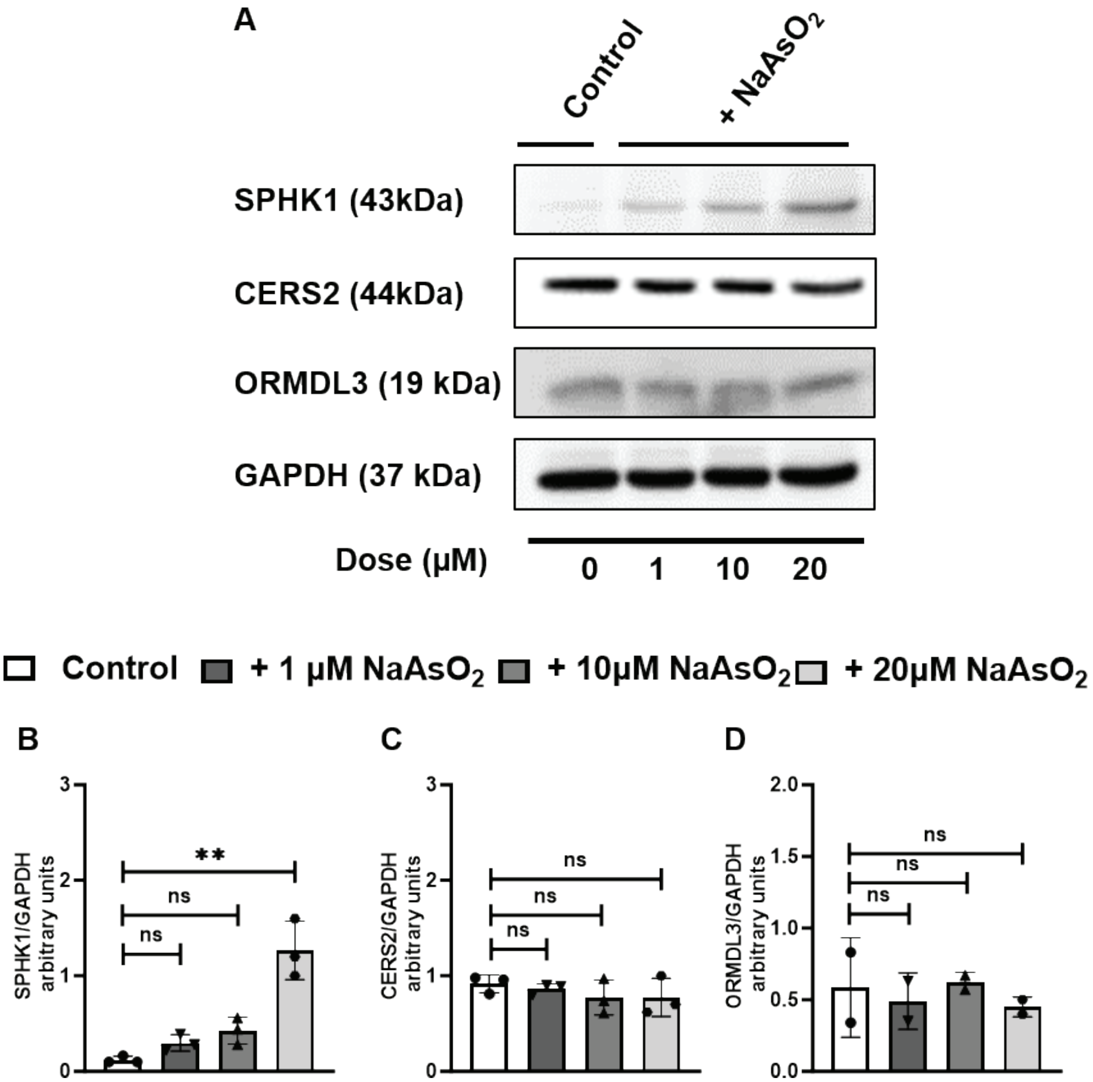

Supplementary Figure S2

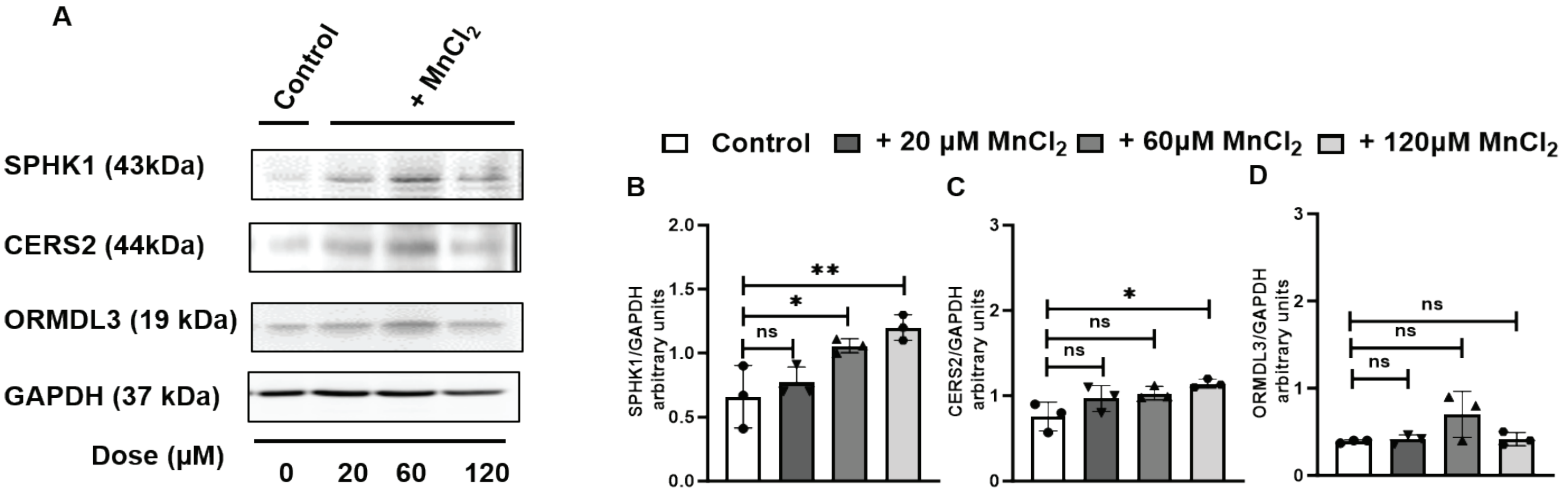

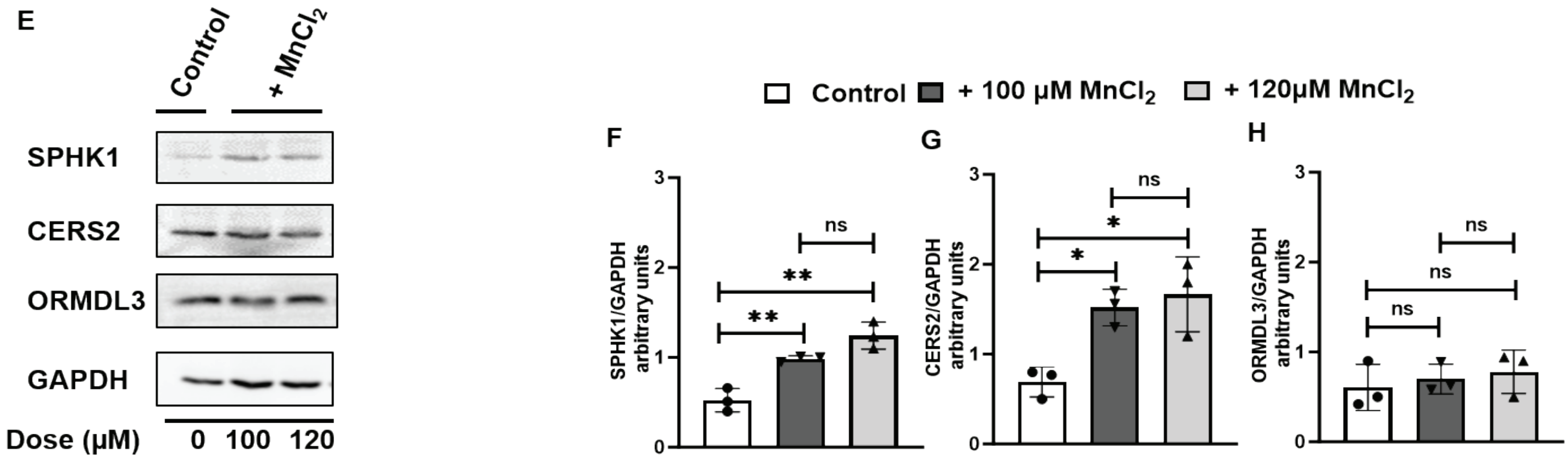

Supplementary Figure S3

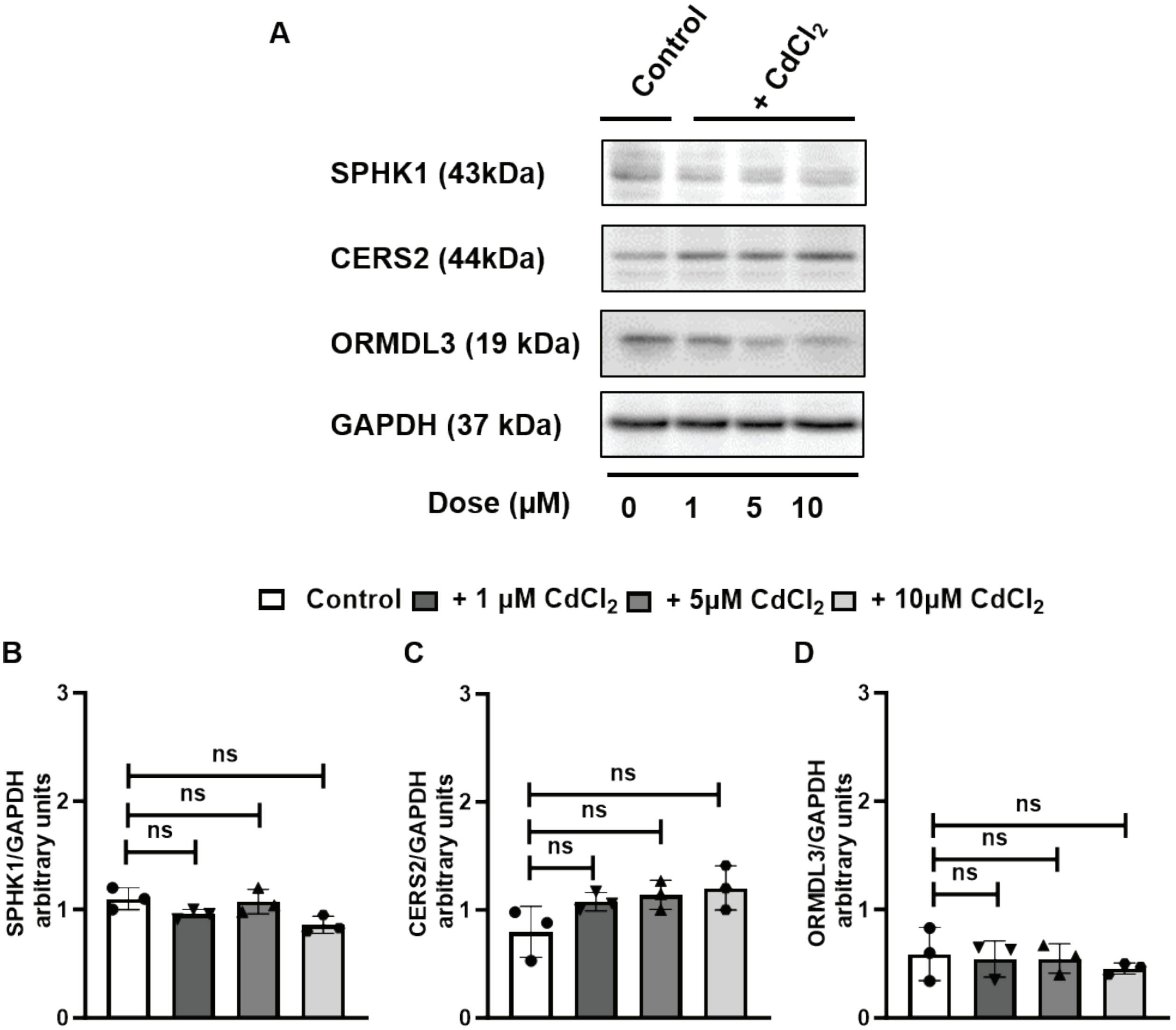

Supplementary Figure S4

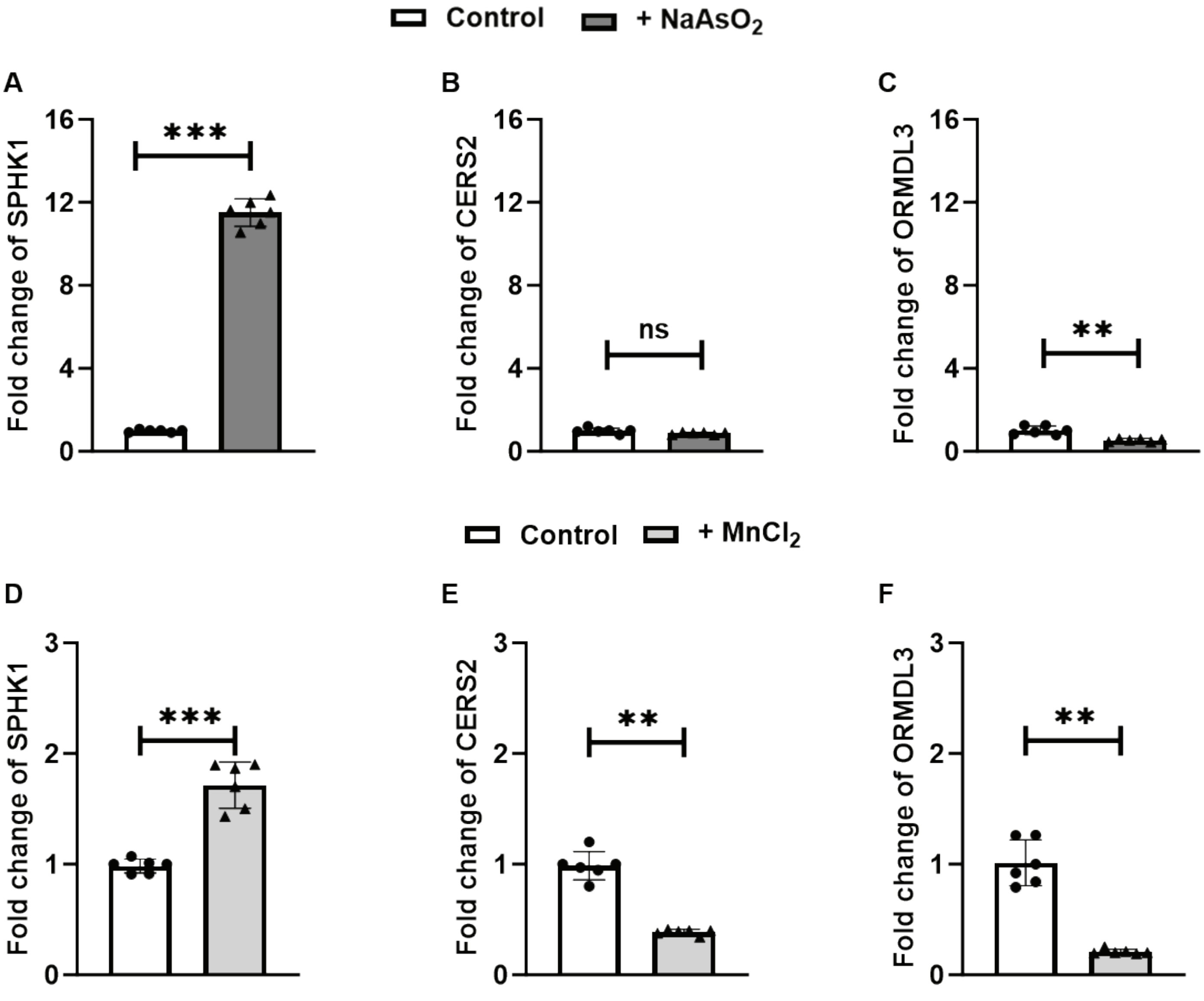

Supplementary Figure S5

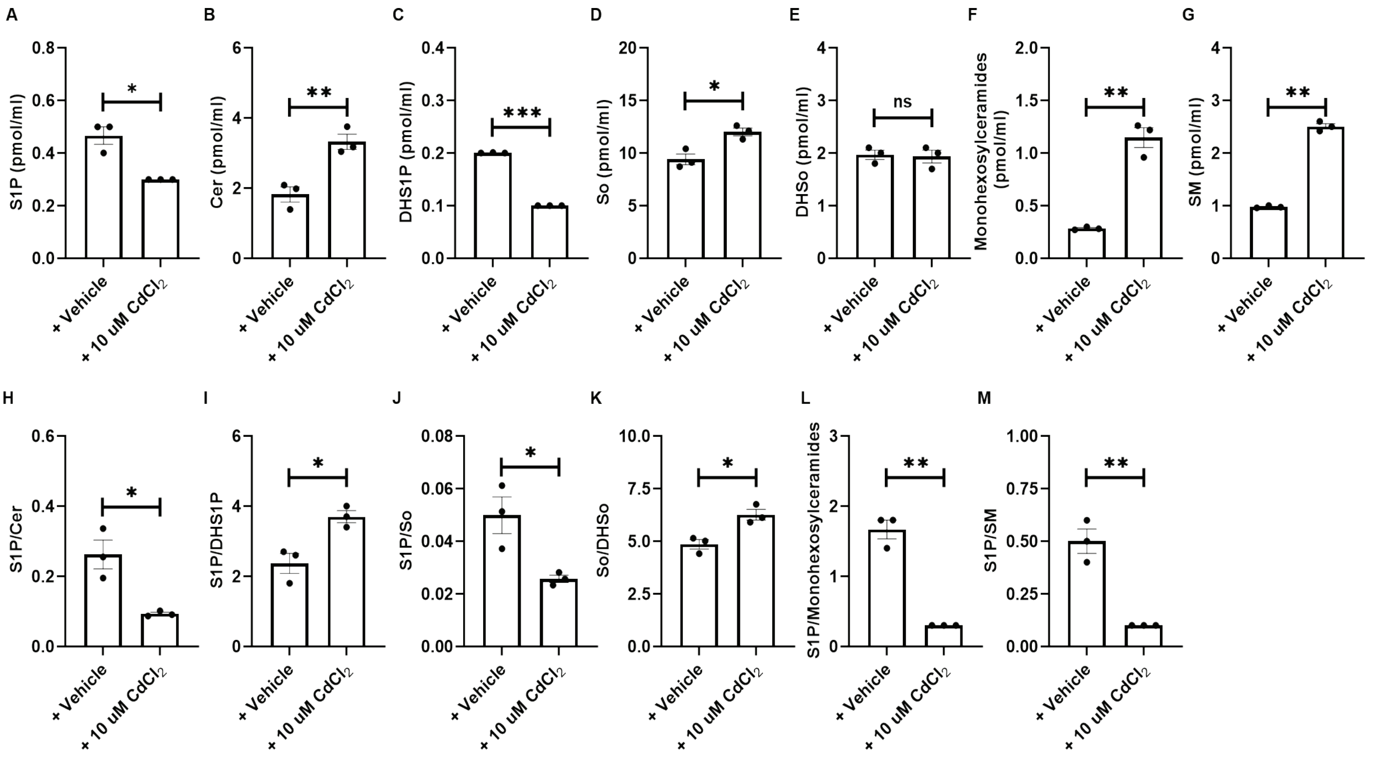

Supplementary Figure S6

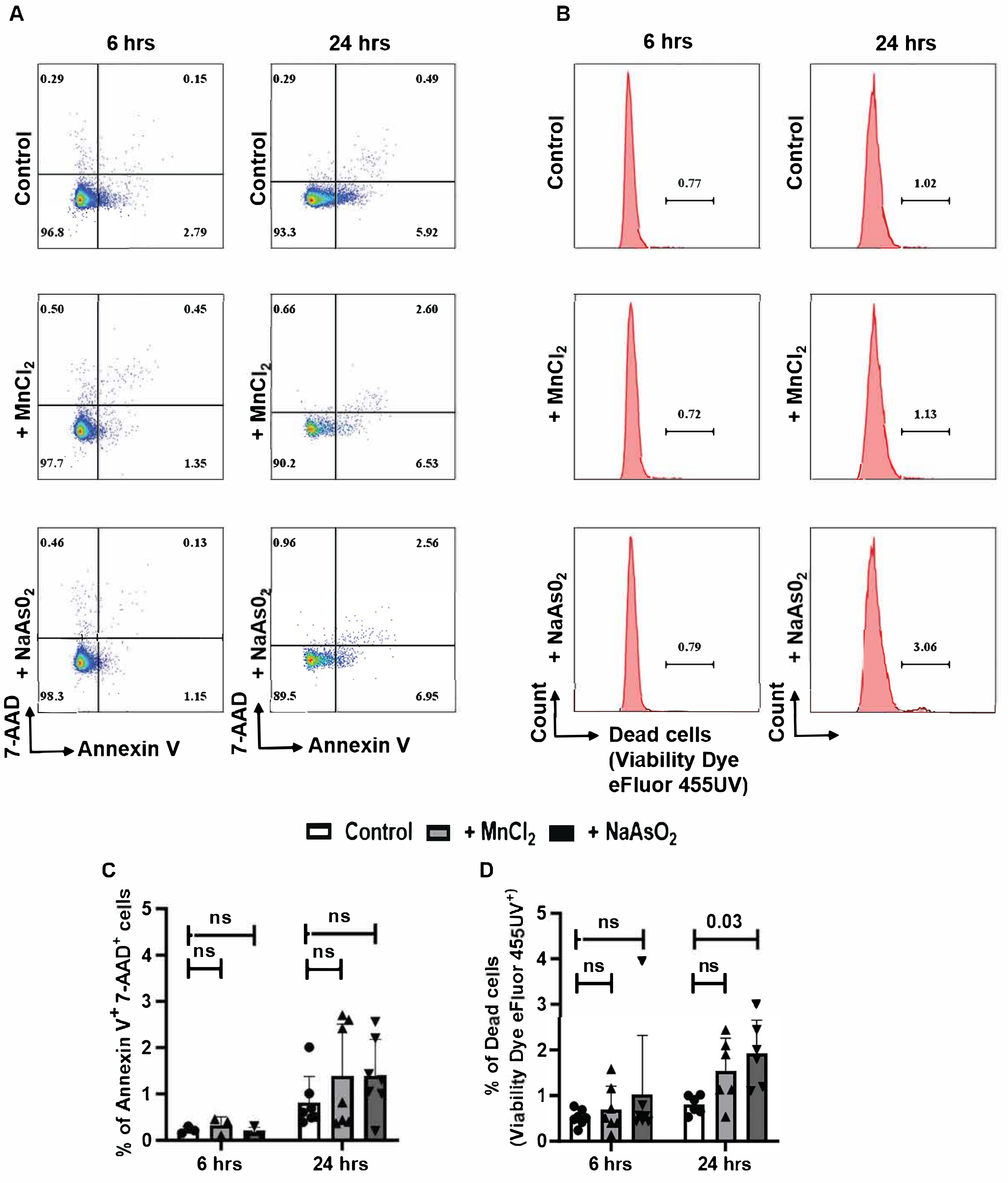

Supplementary Figure S7

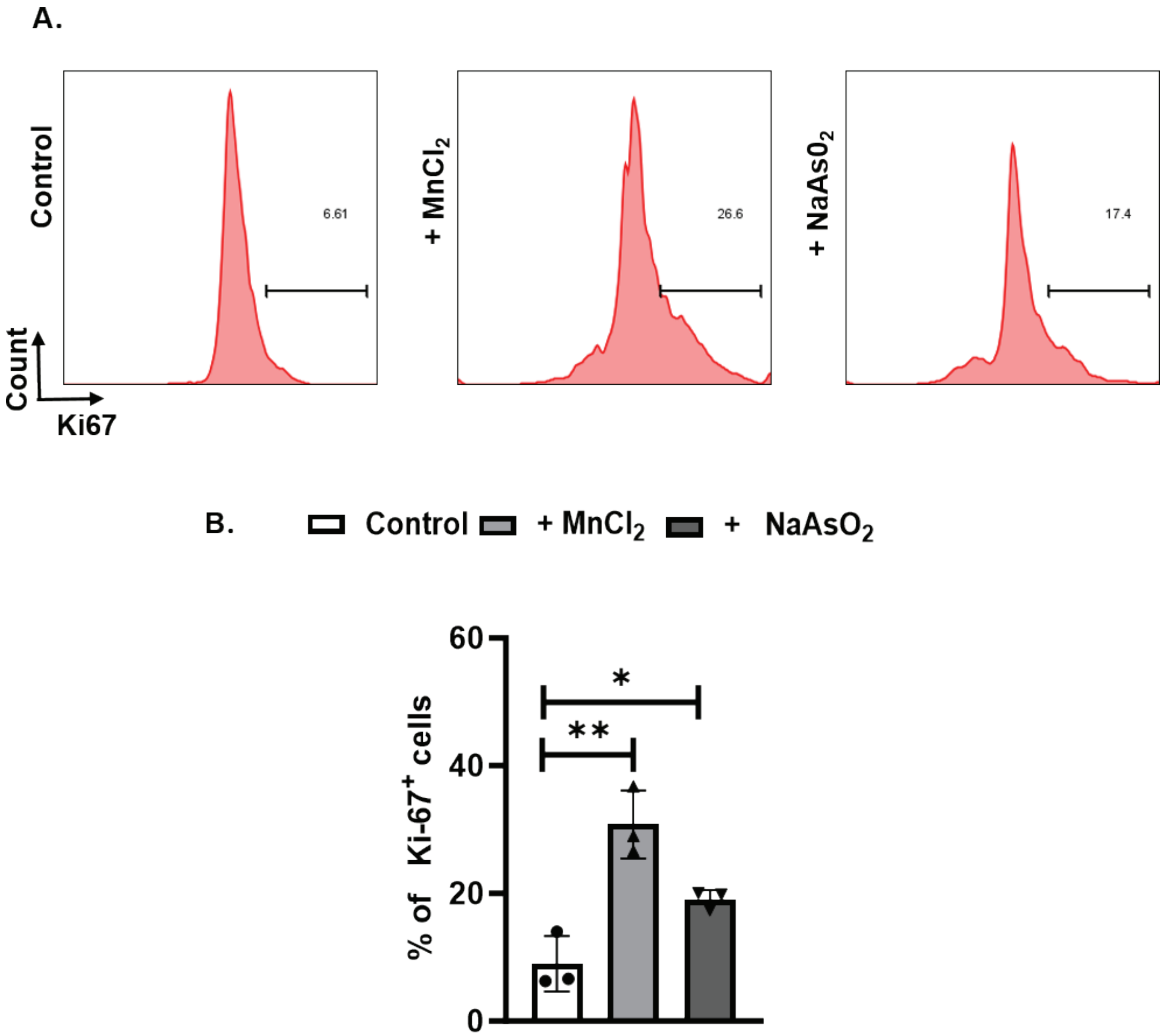

Supplementary Figure S8

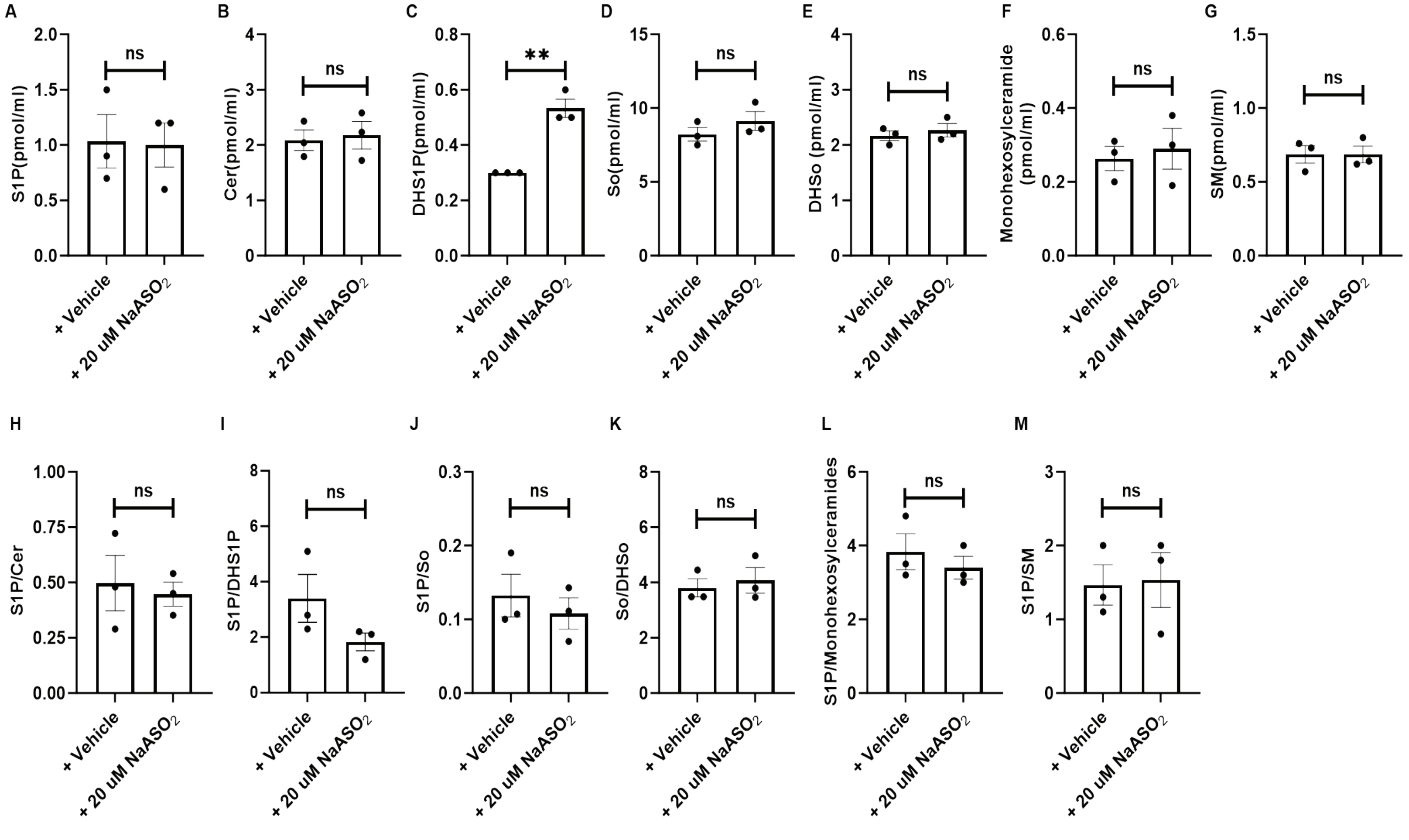

Top 20 Upregualted Oxidative Stress Induced Senescence Genes

$CdCl_2$ vs. all control				$MnCl_2$ vs. all control				$NaAsO_2$ vs. all control			
Target name	Fold change	Pvalue	Adjusted pvalue	Target name	Fold change	Pvalue	Adjusted pvalue	Target name	Fold change	Pvalue	Adjusted pvalue
**IFNB1**	1.91	0.0136	0.0414	FOS	1.6842	0.37967	0.64544	**MAPK10**	1.89	0.0040	0.0363
**CDKN2A**	1.89	0.0489	0.1058	**H3C6**	1.5236	0.22848	0.6008	**IFNB1**	1.61	0.0151	0.0823
**MAPK10**	1.83	0.0008	0.0054	H2AC8	1.5071	0.01625	0.45948	**EZH2**	1.61	0.0018	0.0266
**CBX2**	1.80	0.0053	0.0210	**MAP2K6**	1.4373	0.00225	0.26734	**CDKN2A**	1.58	0.0418	0.1556
**H2AC4**	1.58	0.0080	0.0270	H3C7	1.4233	0.17849	0.6008	**MAP2K6**	1.54	0.0035	0.0363
**H3C6**	1.56	0.0070	0.0248	H2AC19	1.4178	0.06727	0.6008	**H2BC1**	1.53	0.1269	0.3147
**H2AB1**	1.50	0.0303	0.0764	H3C11	1.4151	0.01931	0.45948	**H3C6**	1.51	0.0001	0.0076
**H2BC7**	1.48	0.0118	0.0381	KDM6B	1.3787	0.27976	0.60301	**CBX8**	1.49	0.1356	0.3293
**H3-2**	1.48	0.0227	0.0628	H4C6	1.3669	0.08816	0.6008	H2BU1	1.45	0.0789	0.2183
MAPK11	1.46	0.0094	0.0311	**H2AC4**	1.3559	0.01652	0.45948	**H2AB1**	1.45	0.0493	0.1704
**H2BC1**	1.45	0.0028	0.0153	H2BC8	1.3511	0.10933	0.6008	**E2F2**	1.45	0.0897	0.2426
**CBX8**	1.39	0.1844	0.2849	H2BC21	1.3502	0.0159	0.45948	**H3-2**	1.44	0.1385	0.3295
**E2F3**	1.37	0.1717	0.2688	**MAPK10**	1.3468	0.13116	0.6008	**CBX2**	1.40	0.0544	0.1704
MAPK9	1.37	0.2914	0.4080	H3C3	1.3363	0.26358	0.6008	H2BC3	1.39	0.0408	0.1556
**EZH2**	1.37	0.1405	0.2418	**H3-2**	1.3334	0.04746	0.56481	**H2AC4**	1.39	0.0043	0.0363
AGO4	1.37	0.1933	0.2950	H3C15	1.3189	0.2569	0.6008	H3-3A	1.37	0.2630	0.4671
TNRC6C	1.35	0.1676	0.2659	H3C2	1.3145	0.31156	0.62265	**E2F3**	1.35	0.0926	0.2448
H2BC6	1.35	0.2297	0.3417	**H2BC7**	1.3109	0.0755	0.6008	**E2F1**	1.35	0.0025	0.0328
**H2BU1**	1.32	0.2618	0.3771	**H2BU1**	1.2981	0.16077	0.6008	**H2AC7**	1.32	0.2278	0.4444
**E2F1**	1.29	0.0714	0.1402	**H2AC7**	1.2873	0.04252	0.56221	MOV10	1.30	0.0533	0.1704

Top 20 Downregualted Oxidative Stress Induced Senescence Genes

$CdCl_2$ vs. all control				$MnCl_2$ vs. all control				$NaAsO_2$ vs. all control			
Target name	Fold change	Pvalue	Adjusted pvalue	Target name	Fold change	Pvalue	Adjusted pvalue	Target name	Fold change	Pvalue	Adjusted pvalue
**RPS27A**	-9.47	0.0000	0.0011	**RPS27A**	-1.482	0.10668	0.6008	**RPS27A**	-3.42	0.0159	0.0823
**UBA52**	-7.25	0.0000	0.0005	**UBA52**	-1.397	0.14238	0.6008	**H4C12**	-3.42	0.0003	0.0079
**TXN**	-5.77	0.0000	0.0002	**TXN**	-1.314	0.41485	0.69531	**H4C15**	-3.16	0.0004	0.0079
**H4C12**	-4.44	0.0002	0.0021	**PHC2**	-1.264	0.17797	0.6008	**UBA52**	-3.05	0.1698	0.3795
**FOS**	-4.29	0.0194	0.0564	SUZ12	-1.229	0.04058	0.56221	**JUN**	-2.44	0.0035	0.0363
**JUN**	-4.26	0.0008	0.0054	BMI1	-1.219	0.10112	0.6008	**PHC2**	-2.29	0.0009	0.0150
H2AC19	-3.61	0.0000	0.0005	TNIK	-1.214	0.2679	0.6008	**H4C5**	-2.28	0.0001	0.0076
**MAPKAPK2**	-3.37	0.0001	0.0015	**MAPKAPK2**	-1.197	0.35932	0.63977	**FOS**	-2.26	0.0407	0.1556
**H3-3B**	-3.24	0.0004	0.0030	**H4C12**	-1.192	0.97809	0.97809	**MAP4K4**	-1.96	0.0219	0.1041
H2AZ1	-3.11	0.0001	0.0014	TFDP2	-1.173	0.06843	0.6008	**MAPKAPK2**	-1.84	0.0047	0.0363
**MAP4K4**	-2.88	0.0013	0.0077	**CDK4**	-1.162	0.34824	0.63754	**KDM6B**	-1.82	0.0070	0.0493
**PHC2**	-2.73	0.0004	0.0031	CBX6	-1.161	0.26839	0.6008	**TXN**	-1.75	0.9527	0.9773
UBB	-2.65	0.0009	0.0054	TFDP1	-1.147	0.24657	0.6008	H2AC6	-1.63	0.0040	0.0363
**CDK4**	-2.61	0.0004	0.0030	TNRC6A	-1.145	0.11919	0.6008	H2BC4	-1.61	0.0002	0.0076
MAPK1	-2.37	0.0004	0.0030	**MAP4K4**	-1.129	0.49491	0.75154	**H2BC5**	-1.59	0.0003	0.0076
**H4C15**	-2.34	0.0014	0.0079	MAPKAPK3	-1.115	0.33959	0.63754	H4C4	-1.56	0.0098	0.0650
**KDM6B**	-2.22	0.0035	0.0157	PHC3	-1.113	0.18302	0.6008	MAP2K3	-1.53	0.1073	0.2776
**H2BC5**	-2.20	0.0001	0.0014	PHC1	-1.099	0.17464	0.6008	**H3-3B**	-1.49	0.3414	0.5532
H3-3A	-2.13	0.0003	0.0030	TNRC6B	-1.094	0.27247	0.6008	H2AC20	-1.48	0.0121	0.0759
**H4C5**	-2.06	0.0003	0.0027	H2AZ1	-1.093	0.58234	0.81528	MAPK1	-1.48	0.1153	0.2919

Supplementary Table S1: Top 20 Up- and Down-regulated Reactome Oxidative Stress Induced Senescence Pathway Genes Across HM Exposures. Bold brown text denotes common genes across all three HM exposures, blue bold text denotes common genes with $CdCl_2$ and $NaAsO_2$ exposure, purple bold text denotes common genes with $CdCl_2$ and $MnCl_2$ exposure, andbold green text denotes common genes with $MnCl_2$ and $NaAsO_2$ exposure.

Top 10 Upregualted FOXO-Mediated Oxidative Stress Genes

$CdCl_2$ v all control				$MnCl_2$ v all control				$NaAsO_2$ v all control			
Target name	Fold change	Pvalue	Adjusted pvalue	Target name	Fold change	Pvalue	Adjusted pvalue	Target name	Fold change	Pvalue	Adjusted pvalue
**POMC**	1.64	0.0049	0.0344	**AGRP**	1.3491	0.09203	0.61816	**G6PC1**	1.64	0.0010	0.0147
**AGRP**	1.62	0.0160	0.0579	**POMC**	1.3016	0.05204	0.50301	**AGRP**	1.58	0.0388	0.1023
TRIM63	1.61	0.0221	0.0612	**PLXNA4**	1.2845	0.02801	0.50301	**POMC**	1.57	0.0537	0.1198
**PPARGC1**	1.55	0.0681	0.1162	**IGFBP1**	1.2253	0.11243	0.61816	**IGFBP1**	1.55	0.0001	0.0017
**NPY**	1.53	0.0071	0.0344	NR3C1	1.1667	0.24609	0.71367	**RETN**	1.54	0.0122	0.0625
**GCK**	1.47	0.0759	0.1223	FBXO32	1.1583	0.1279	0.61816	**PLXNA4**	1.44	0.0298	0.0864
**G6PC1**	1.46	0.1240	0.1710	**RETN**	1.1485	0.30386	0.77898	**PCK1**	1.43	0.0151	0.0625
INS	1.44	0.0275	0.0613	**NPY**	1.1314	0.49941	0.77898	**NPY**	1.35	0.1134	0.2055
**RETN**	1.43	0.0241	0.0612	**GCK**	1.1196	0.46604	0.77898	**PPARGC1**	1.32	0.1075	0.2055
**PCK1**	1.41	0.0578	0.1049	**PCK1**	1.1163	0.52015	0.77898	ABCA6	1.30	0.0584	0.1210

Top 10 Downregualted FOXO-Mediated Oxidative Stress Genes

$CdCl_2$ v all control				$MnCl_2$ v all control				$NaAsO_2$ v all control			
Target name	Fold change	Pvalue	Adjusted pvalue	Target name	Fold change	Pvalue	Adjusted pvalue	Target name	Fold change	Pvalue	Adjusted pvalue
**SOD2**	-14.45	0.0061	0.0344	**CAT**	-1.373	0.04564	0.50301	**SOD2**	-4.16	0.0465	0.1123
**FOXO3**	-2.04	0.0023	0.0344	**FOXO3**	-1.136	0.36517	0.77898	**FOXO3**	-1.68	0.0222	0.0717
**NR3C1**	-1.90	0.0068	0.0344	**SMAD2**	-1.128	0.24233	0.71367	**NR3C1**	-1.43	0.0103	0.0625
**SMAD4**	-1.81	0.0060	0.0344	**SMAD4**	-1.127	0.23177	0.71367	**SMAD2**	-1.35	0.1588	0.2558
**HDAC2**	-1.66	0.0471	0.0977	**SREBF1**	-1.107	0.21934	0.71367	**SMAD4**	-1.28	0.0151	0.0625
**SMAD2**	-1.62	0.0140	0.0579	**HDAC2**	-1.079	0.55231	0.77898	**CAT**	-1.21	0.5110	0.7285
**CAT**	-1.40	0.0253	0.0612	**SIN3A**	-1.07	0.64468	0.77898	**FOXO1**	-1.21	0.0210	0.0717
**SREBF1**	-1.40	0.0193	0.0612	**FOXO1**	-1.053	0.4928	0.77898	**SREBF1**	-1.13	0.1374	0.2344
**SIN3A**	-1.17	0.1131	0.1640	SIRT3	-1.049	0.44294	0.77898	**HDAC2**	-1.11	0.8346	0.8964
**SMAD3**	-1.09	0.1297	0.1710	ATXN3	-1.047	0.61791	0.77898	**SMAD3**	-1.11	0.1945	0.2968

Supplementary Table S2: Top 10 Up- and Down-regulated Reactome FOXO-Mediated Oxidative Stress Pathway Genes across HM Exposures. Bold brown text denotes common genes across all three HM exposures, blue bold text denotes common genes with $CdCl_2$ and $NaAsO_2$ exposure, purple bold text denotes common genes with $CdCl_2$ and $MnCl_2$ exposure, andbold green text denotes common genes with $MnCl_2$ and $NaAsO_2$ exposure.

Supplementary Figure 9

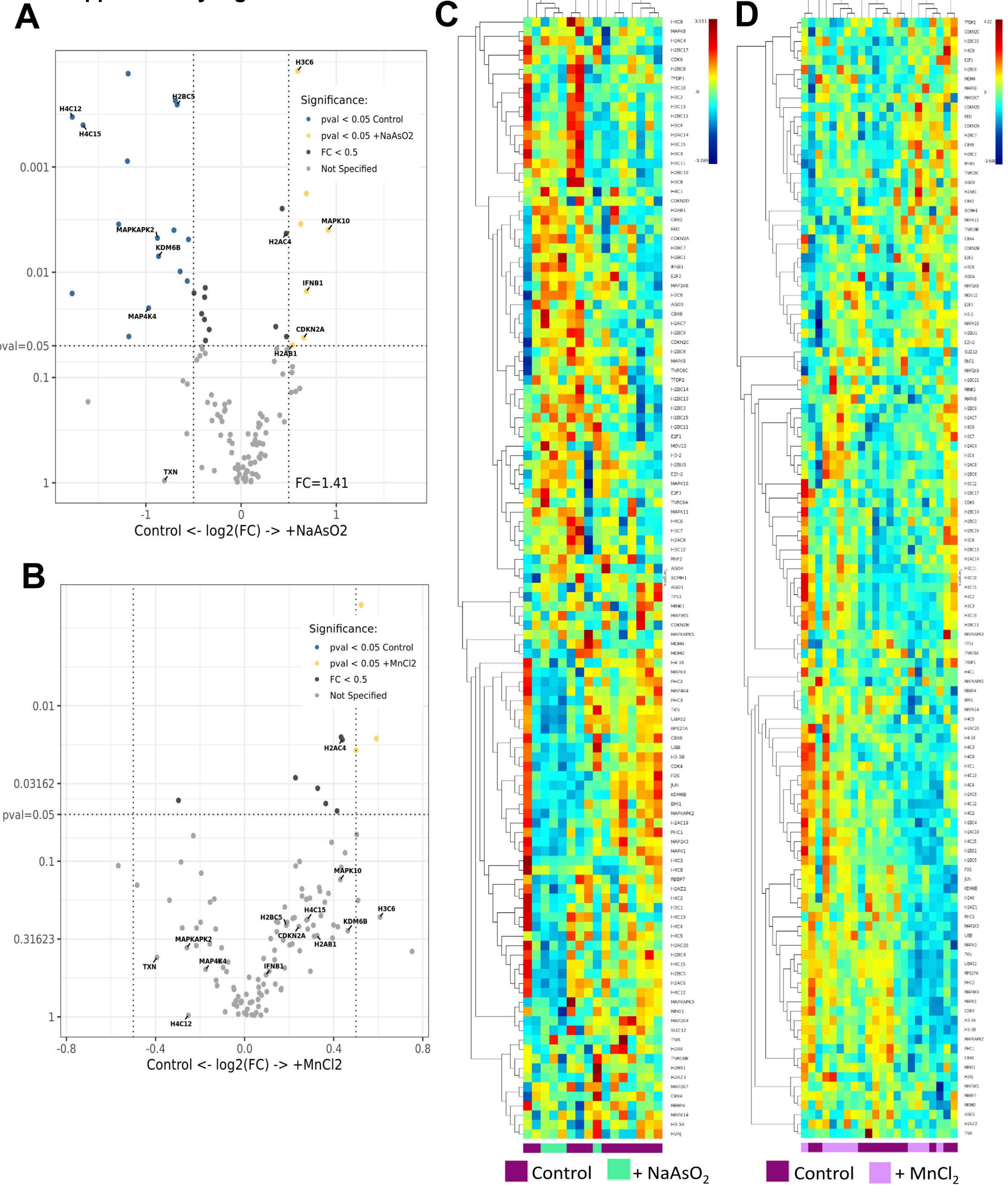

Supplementary Figure S10

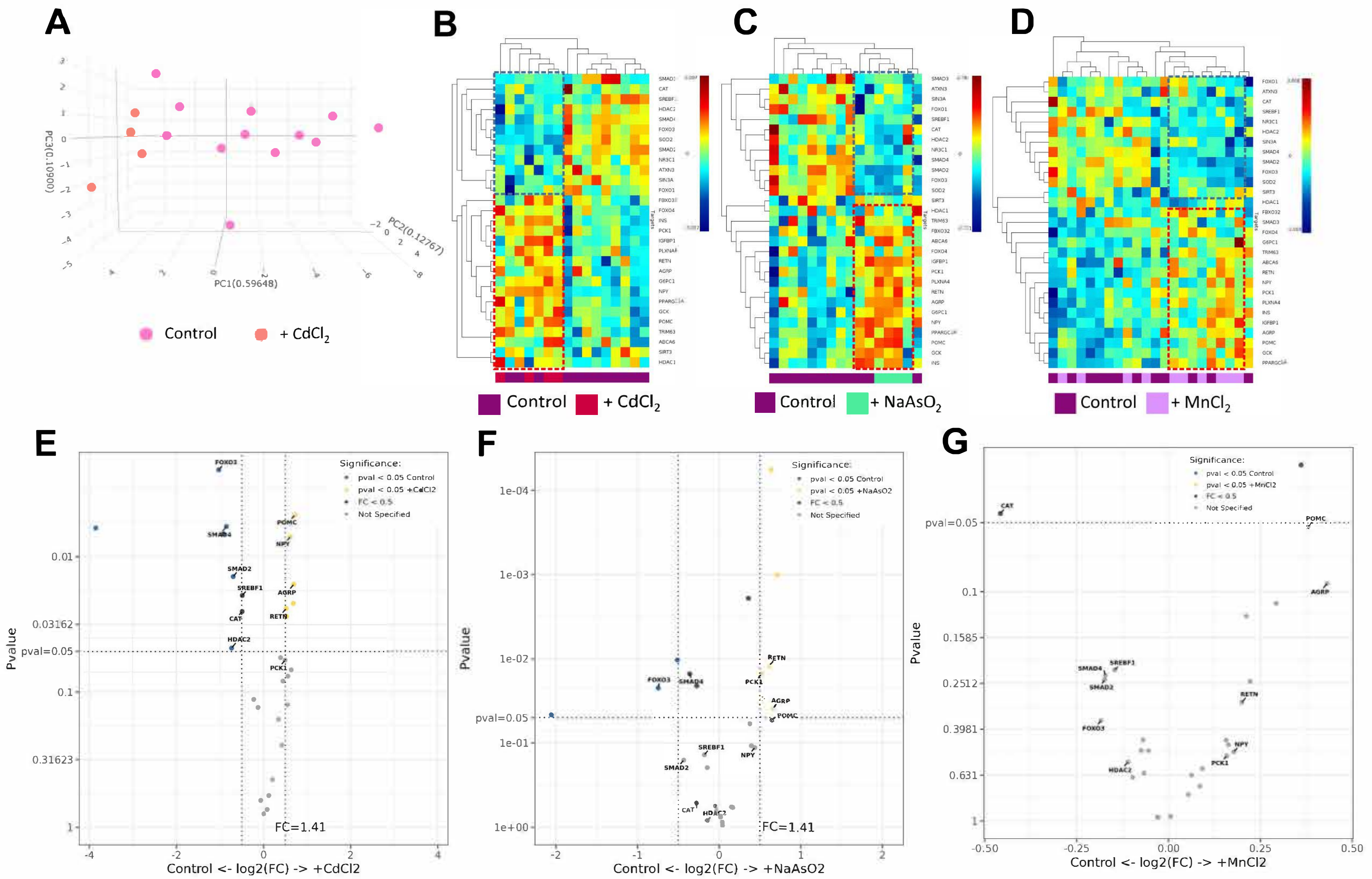

Supplementary Figure S11

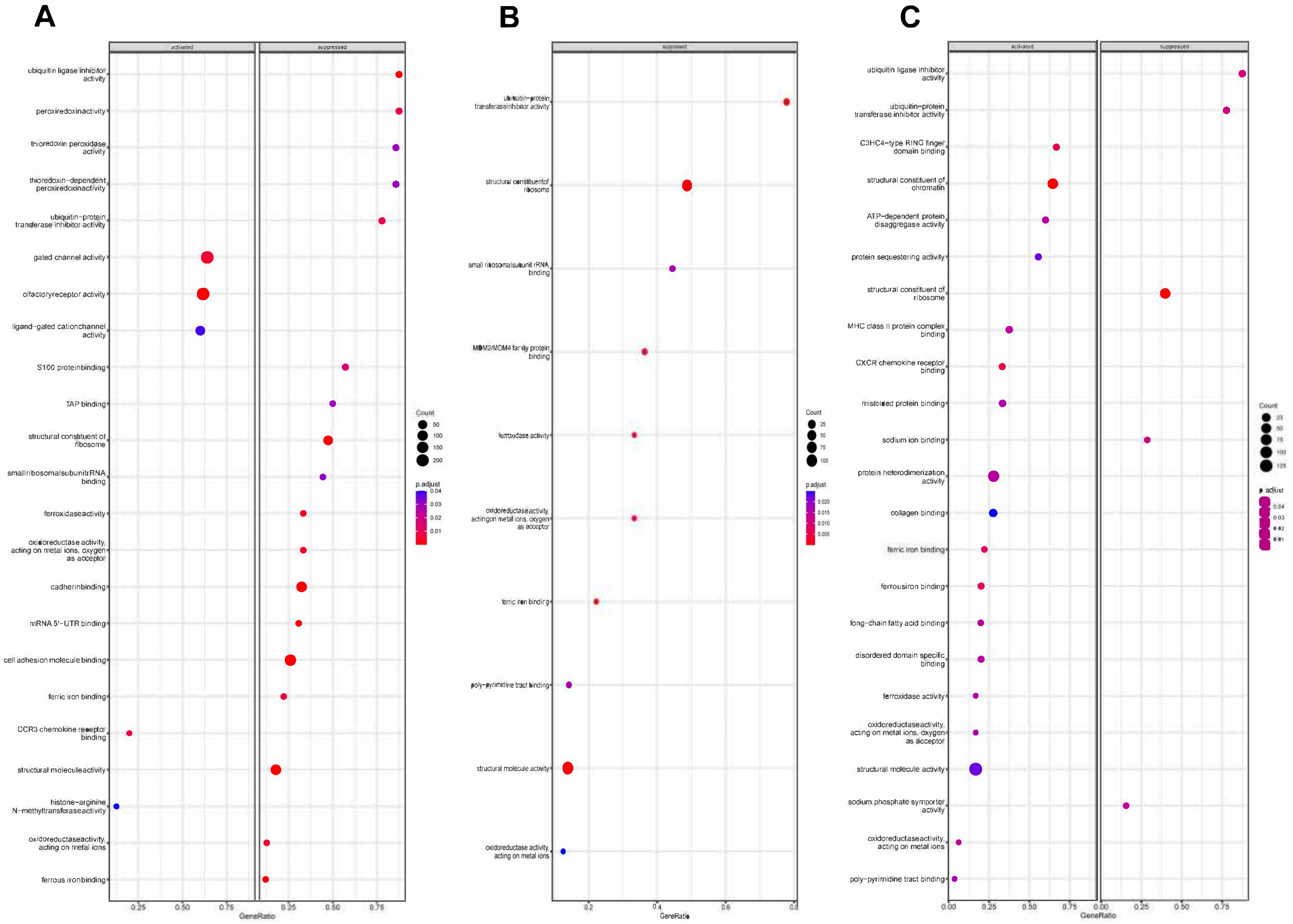

Supplementary Figure S12

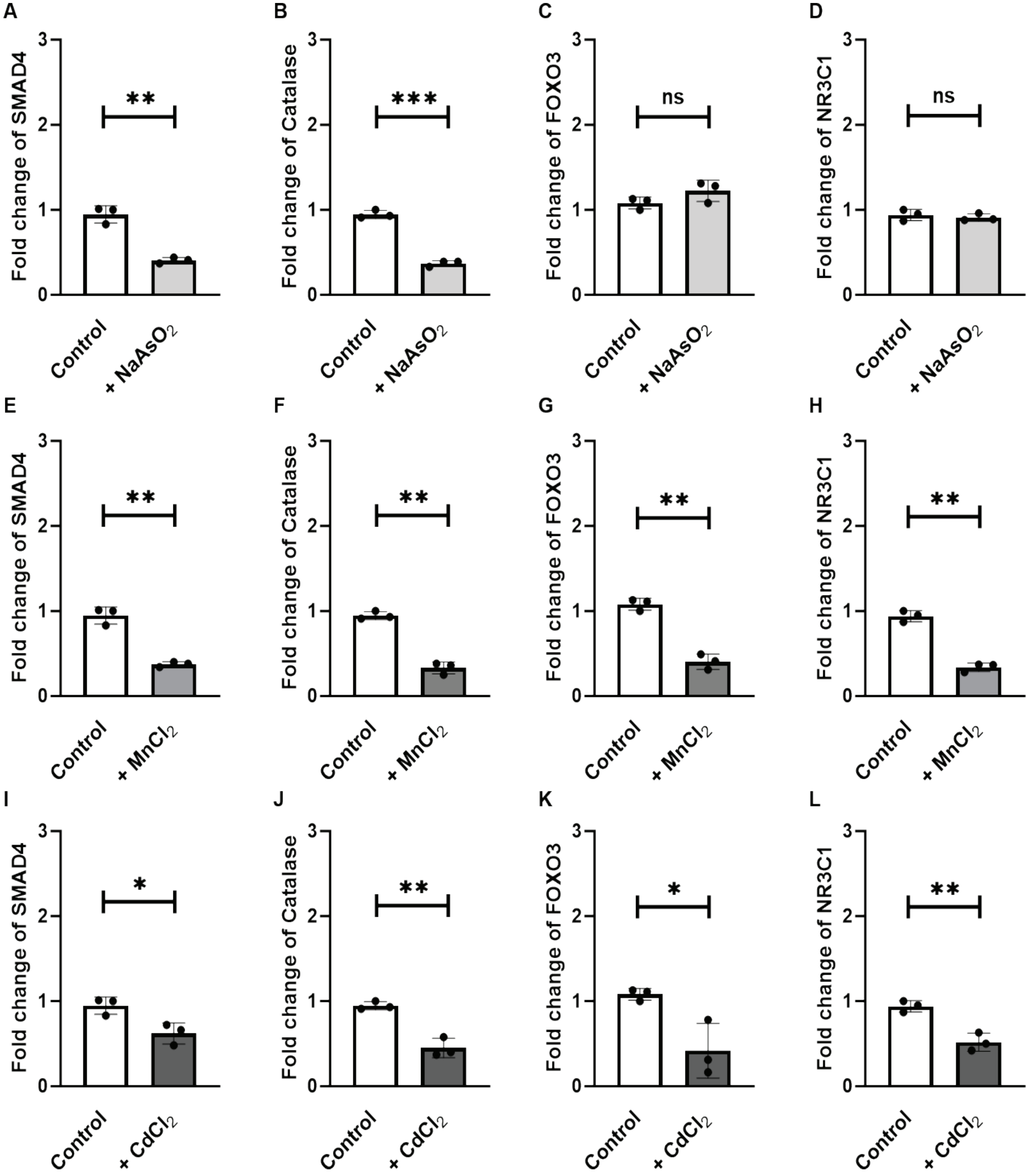

Supplementary Figure S13

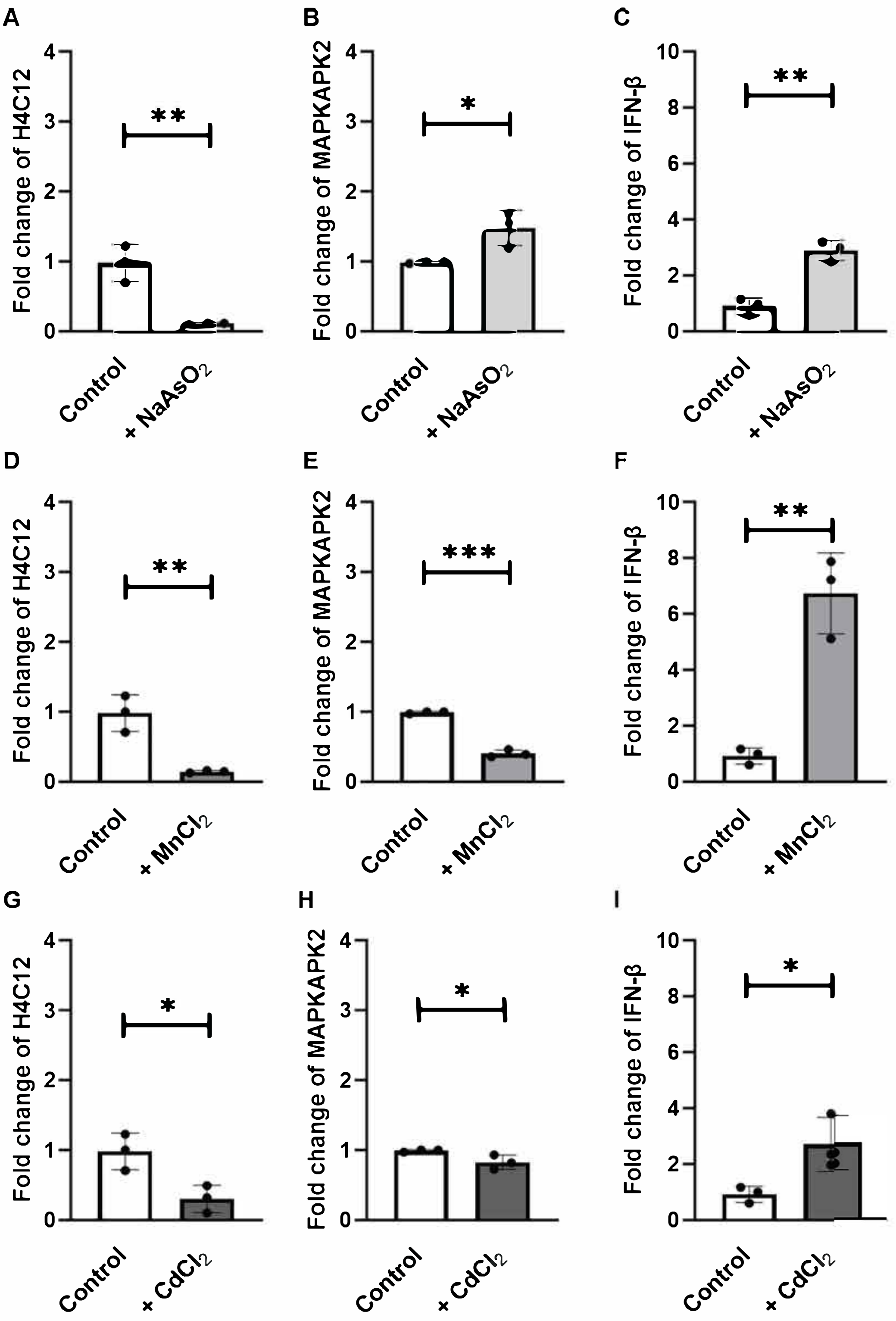

Supplement: Supplementary file 1 [file antioxidants-13-00978-s001.zip › antioxidants-3076580- Supplementary-update.pdf]
