# Supplementary material for: Heavy Metal Exposure-Mediated Dysregulation of Sphingolipid Metabolism
Source: Antioxidants (Basel). 2024 Aug 12;13(8):978. doi: 10.3390/antiox13080978 (PMC13317051; doi:10.3390/antiox13080978)
Supplement: Supplementary file 1 [file antioxidants-13-00978-s001.zip › antioxidants-3076580- Supplementary Figure Legends-update.pdf]

Supplementary Figure S1. Dose-dependent alteration in sphingolipid metabolic pathways in $NaAsO_2$-exposed lung epithelial cells. (A) Immunoblot analysis of controls and different doses (1 μM, 10 μM, and 20 μM) of $NaAsO_2$-exposed BEAS-2B cells for SPHK1, CERS2, ORMDL3, and GAPDH expression. (B-D) Densitometry analysis of controls and different doses of $NaAsO_2$-exposed BEAS-2B cells, illustrating SPHK1 expression (B), CERS2 expression (C), and ORMDL3 expression (D). One-way ANOVA with Tukey's multiple comparisons test for data with more than two groups was performed to determine the statistical significance at alpha 0.05. Each bar represents the mean ± SD of three independent experiments. * $p < 0.05$, ** $p < 0.01$; *** $p < 0.001$.

Supplementary Figure S2. Dose-dependent alteration in sphingolipid metabolic pathways in $MnCl_2$-exposed lung epithelial cells. (A) Immunoblot analysis of controls and different doses (20 μM, 60 μM, and 120 μM) of $MnCl_2$-exposed BEAS-2B cells for SPHK1, CERS2, ORMDL3, and GAPDH expression. (B-D) Densitometry analysis of controls and different doses of $MnCl_2$-exposed BEAS-2B cells illustrating SPHK1 expression (B), CERS2 expression (C), and ORMDL3 expression (D). (E) Immunoblot analysis of controls and different doses (100 μM and 120 μM) of $MnCl_2$-exposed BEAS-2B cells illustrating SPHK1, CERS2, ORMDL3, and GAPDH expression. (F–H) Densitometry analysis of controls and different doses of $MnCl_2$-exposed BEAS-2B cells for SPHK1 expression (F), CERS2 expression (G), and ORMDL3 expression (H). One-way ANOVA with Tukey's multiple comparisons test for data with more than two groups was performed to determine the statistical significance at alpha 0.05. Each bar represents the mean ± SD of 3 independent experiments. * $p < 0.05$, ** $p < 0.01$; *** $p < 0.001$.

Supplementary Figure S3. Dose-dependent alteration in sphingolipid metabolic pathways in $CdCl_2$-exposed lung epithelial cells. (A) Immunoblot analysis of controls and different doses (1 μM, 5 μM, and 10 μM) of $CdCl_2$-exposed BEAS-2B cells for SPHK1, CERS2, ORMDL3, and GAPDH expression. (B–D) Densitometry analysis of controls and different doses of $CdCl_2$-exposed BEAS-2B cells for SPHK1 expression (B), CERS2 expression (C), and ORMDL3 expression (D). One-way ANOVA with Tukey's multiple comparisons test for data with more than two groups was performed to determine the statistical significance at alpha 0.05. Each bar represents the mean ± SD of 3 independent experiments. * $p < 0.05$, ** $p < 0.01$; *** $p < 0.001$.

Supplementary Figure S4. Alteration in sphingolipid metabolic pathways regulating enzyme transcript levels in HM-exposed lung epithelial cells. (A–C) Fold change in enzyme expression in $NaAsO_2$-exposed BEAS-2B cells SPHK1 (A), CERS2 (B), and ORMDL3 (C). (D–F) Fold change in enzyme expression in $MnCl_2$-exposed BEAS-2B cells, SPHK1 (D), CERS2 (E), and ORMDL3 (F). A two-tailed unpaired Student's *t*-test was performed to determine statistical significance at alpha 0.05. Each bar represents the mean ± SD of 3 independent experiments. * $p < 0.05$, ** $p < 0.01$; *** $p < 0.001$.

Supplementary Figure S5. Alteration of sphingolipid metabolites in $CdCl_2$-exposed lung epithelial cells. (A–G) Levels of S1P (A), Cer (B), DHSIP (C), So (D), DHSo (E), monohexosylceramides (F), and SM (G). (H–M) The ratio of abundance of S1P/Cer (H), S1P/DHS1P (I), S1P/So (J), So/DHSo (K), S1P/monohexosylceramides (L), and S1P/SM (M). A two-tailed unpaired Student's *t*-test was performed to determine statistical significance at alpha 0.05. Each bar represents the mean ± SD of 3 independent experiments. * $p < 0.05$, ** $p < 0.01$; *** $p < 0.001$.

Supplementary Figure S6. Quantitation of apoptosis and cell viability in lung epithelial cells exposed to HMs ($MnCl_2$ and $NaAsO_2$). (A,B) Representative image of controls and HM-exposed cells for Annexin V^+ $7AAD^+$ (A), Viability Dye eFlour $455UV^+$ (B). (C,D). Graphical representation of controls and HM-exposed cells for Annexin V^+ $7AAD^+$ (C) and Viability Dye eFlour 455 UV^+ (D). Two-way ANOVA with Sidak's multiple comparisons test for data with more than two groups was performed to determine the statistical significance at alpha 0.05. Each bar represents the mean ± SD of 3 independent experiments. * $p < 0.05$, ** $p < 0.01$; *** $p < 0.001$.

Supplementary Figure S7. Quantitation of proliferation of HM ($MnCl_2$ and $NaAsO_2$) exposed lung epithelial cells. (A) Representative image of controls and HM-exposed cells for $Ki67^+$. (B) Graphical representation of controls and HM-exposed cells for $Ki67^+$. One-way ANOVA with Tukey's multiple comparisons test for data with more than two groups was performed to determine the statistical significance at alpha 0.05. Each bar represents the mean ± SD of 3 independent experiments. * $p < 0.05$, ** $p < 0.01$; *** $p < 0.001$.

Supplementary Figure S8. Alteration of sphingolipid metabolites in $NaAsO_2$-exposed lung tissues. (A–G). Levels of S1P (A), Cer (B), DHSIP (C), So (D), DHSo (E), monohexosylceramides (F), and SM (G). (H–M) The ratio of abundance of S1P/Cer (H), S1P/DHS1P (I), S1P/So (J), So/DHSo (K), S1P/monohexosylceramides (L), and S1P/SM (M). A two-tailed unpaired Student's *t*-test was performed to determine statistical significance at alpha 0.05. Each bar represents the mean ± SD of 3 independent experiments. * $p < 0.05$, ** $p < 0.01$; *** $p < 0.001$.

Supplementary Table S1. Top 20 upregulated and downregulated Reactome Oxidative Stress-Induced Senescence Pathway Genes across HM Exposures. Bold brown text denotes common genes across all three HM exposures, blue bold text denotes common genes with $CdCl_2$ and $NaAsO_2$ exposure, purple bold text denotes common genes with $CdCl_2$ and $MnCl_2$ exposure, and bold green text denotes common genes with $MnCl_2$ and $NaAsO_2$ exposure.

Supplementary Table S2. Top 10 upregulated and downregulated Reactome FOXO-Mediated Oxidative Stress Pathway genes across HM exposures. Bold brown text denotes common genes across all three HM exposures, blue bold text denotes common genes with $CdCl_2$ and $NaAsO_2$ exposure, purple bold text denotes common genes with $CdCl_2$ and $MnCl_2$ exposure, and bold green text denotes common genes with $MnCl_2$ and $NaAsO_2$ exposure.

Supplementary Figure S9. Reactome FOXO Mediated Oxidative Stress Pathway changes following HM exposure. (A) PCA plot of $CdCl_2$-exposed and control tissues following spatial transcriptomics shows differential clustering of exposed and control ROIs when FOXO Mediated Oxidative Stress genes were compared. (B–D). Clustered heatmap of FOXO Mediated Oxidative Stress genes showing ROIs from $CdCl_2$-exposed and control tissues (B), $NaAsO_2$-exposed and control tissues (C), and $MnCl_2$-exposed and control tissues (D). The red box shows genes predominately upregulated with $NaAsO_2$ exposure and the blue box shows genes predominately downregulated with $NaAsO_2$ exposure. (E–G) Volcano plots showing differentially regulated FOXO Mediated Oxidative Stress genes following $CdCl_2$ exposure (E), $NaAsO_2$ exposure (F), or $MnCl_2$ exposure (G).

Supplementary Figure S10. Reactome Oxidative Stress-Induced Senescence Pathway changes following $NaAsO_2$ or $MnCl_2$ exposure. (A,B) Clustered heatmap of Oxidative Stress-Induced Senescence genes showing ROIs from $NaAsO_2$-exposed and control tissues (A), and $MnCl_2$-exposed and control tissues (B). (C,D) Volcano plots showing differentially regulated Oxidative Stress-Induced Senescence genes following $NaAsO_2$ exposure (C) or $MnCl_2$ exposure (D).

Supplementary Figure S11: Untargeted Gene Set Enrichment Analysis shows suppressed Redoxin and Oxidase Pathway activity following HM exposure. (A–C). Activated and suppressed Molecular Functions Gene Set Enrichment Analysis following $CdCl_2$ (A), $NaAsO_2$ (B), or $MnCl_2$ (C) exposures when compared to controls.

Supplementary Figure S12. Alteration in oxidative stress-regulating enzymes transcript level in HM-exposed lung epithelial cells. (A–D) Fold change in enzyme expression in $NaAsO_2$-exposed BEAS-2B cells SMAD4 (A), Catalase (B), FOXO3 (C), and NR3C1 (D). (E–H) Fold change in enzyme expression in $MnCl_2$-exposed BEAS-2B cells, SMAD4 (E), Catalase (F), FOXO3 (G), and NR3C1 (H). (I–L) Fold change in enzyme expression in $CdCl_2$-exposed BEAS-2B cells, SMAD4(I), Catalase (J), FOXO3 (K), and NR3C1 (L). A two-tailed unpaired Student's *t*-test was performed to determine statistical significance at alpha 0.05. Each bar represents the mean ± SD of 3 independent experiments. * $p < 0.05$, ** $p < 0.01$; *** $p < 0.001$.

Supplementary Figure S13. Alteration in oxidative stress-induced senescence gene expression in HM-exposed lung epithelial cells. (A–C) Fold change in gene expression in $NaAsO_2$-exposed BEAS-2B cells H4C12 (A), MAPKAPK2 (B), and IFN-β (C). (D–F) Fold change in gene expression in $MnCl_2$-exposed BEAS-2B cells, H4C12 (D), MAPKAPK2 (E), and IFN-β (F). (G–I) Fold change in enzyme expression in $CdCl_2$-exposed BEAS-2B cells, H4C12 (G), MAPKAPK2 (H), and IFN-β (I). A two-tailed unpaired Student's *t*-test was performed to determine statistical significance at alpha 0.05. Each bar represents the mean ± SD of 3 independent experiments. * $p < 0.05$, ** $p < 0.01$; *** $p < 0.001$.
